# STAR-RIS enabled covert integration of sensing communication and over-the-air computing with analysis and optimization

**DOI:** 10.1038/s41598-025-04173-3

**Published:** 2025-05-30

**Authors:** Miao Zhang, Chao Wang, Tianyu Ren, Jing Li

**Affiliations:** State Grid Beijing Electric Power Company Electric Power Research Institute, Beijing, China

**Keywords:** Electrical and electronic engineering, Information technology

## Abstract

This paper addresses the challenges of security and reliability in simultaneously transmitting and reflecting reconfigurable intelligence surface (STAR-RIS) assisted integrated sensing, communication, and computation over-the-air (ISCCO) system. We investigate the trade-offs between covert communication, covert sensing, and Over-the-Air Computation (AirComp) reliability in scenarios involving both communication and sensing wardens. Specifically, to meet covert performance and reliability requirements, we derive covert rates for communication, sensing, and air computing under the assumption of perfect channel state information (CSI) by the wardens. The radar sensing Cramér-Rao bound (CRB) and AirComp mean square error (MSE) are used to represent sensing and computing performance. Building on this, we propose an optimization framework to minimize the error probability of AirComp while satisfying the covert communication and covert sensing requirements as well as the radar sensing CRB constraints. To solve the inherently non-convex optimization problem, we propose a smoothed exact penalty algorithm with a twice continuously differentiable property. This approach involves the joint optimization of data transmission beam-forming, radar sensing beamforming, data aggregation beamforming, and the STAR-RIS matrix. The problem is reformulated into a difference of convex functions form to search for a local optimum. Simulations demonstrate that our algorithm surpasses benchmark schemes in convergence and robustness.

## Introduction

The sixth generation (6G) mobile communication system is expected to play a pivotal role in fields such as autonomous vehicles, extended reality (XR), artificial intelligence (AI), smart cities, and digital twins^1^. These applications require extensive data collection, transmission, and processing. However, in traditional approaches, these three operations are performed independently, increasing data latency and overall delay, while also leading to increased hardware resource and energy consumption and exposing the system to greater risks of security vulnerabilities^2,3^. To address these challenges, the ISCCO has been proposed^4^. Additionally, the introduction of STAR-RIS technology has significantly enhanced the security performance, energy efficiency, and resource utilization of ISCCO systems. STAR-RIS optimizes the wireless propagation environment by adjusting the phase of reflective and transmissive elements, thereby holistically improving the overall performance of ISCCO systems^5,6^. Furthermore, recent research has shown that integrating ISAC pilot designs^7^ can improve channel estimation accuracy, which is critical for reliable STAR-RIS-assisted ISCCO operations. In addition, the impact of low dynamic range constraints on RIS-aided ISAC systems, as discussed in 7, is directly relevant to the practical deployment of STAR-RIS-assisted covert ISCCO systems.

However, the complexity and uncertainty of channels during sensing and communication inevitably lead to security concerns, such as leakage of privacy data from model parameters or eavesdropping attacks^9^. To mitigate these issues, Physical Layer Security (PLS) technologies have emerged as a promising solution^10,11^. Although conventional PLS methods can partially inhibit eavesdroppers, they are insufficient to fully address privacy concerns related to data transmission in ISCCO networks^12^. To this end, covert communication, covert sensing, and covert computation have gained increasing attention, aiming to ensure that the transmission or sensing activity itself remains undetectable. In this paper, we define covert operations in terms of a detection probability threshold, whereby a communication or sensing attempt is deemed covert if the probability of being detected by an adversary remains below a predefined threshold $\epsilon$, typically close to 0.5. This formalization allows us to quantitatively analyze and design systems that satisfy strict concealment constraints while performing their intended functions. For example, in certain scenarios, such as private or military communications, it is critical to protect the mere existence of the transmission, ensuring that it remains undetectable or concealed^13^. Although previous studies^14–16^ have explored covert operations in ISCCO, they have not considered more complex scenarios involving STAR-RIS-assisted covert ISCCO systems in the presence of eavesdroppers.

In military communications, the system’s ability to ensure covertness (via constraints like $\mathscr {D}\left( \mathbb {P}_0 | \mathbb {P}_1\right) _C \le 2\varepsilon _C^2$) while maintaining reliable computation (via AirComp MSE minimization) makes it ideal for secure battlefield networks, where undetected data aggregation is critical for tactical operations, especially in adversarial environments where joint communication, sensing, and jamming are increasingly vital, as explored in recent studies^17–19^. For instance, 17 demonstrates the use of RSMA to manage suspicious communications, while 18 and 19 address cognitive jamming and sensing-resistant attacks, highlighting the need for such integrated approaches in hostile settings. In cognitive radio networks, the joint optimization of sensing and communication can enhance spectrum efficiency, allowing secondary users to perform covert sensing and computation without interfering with primary users, thus improving spectrum sharing in dynamic environments. For IoT deployments, such as smart cities or industrial automation, the system’s low-latency over-the-air computation and STAR-RIS-enabled signal enhancement can support massive device connectivity while ensuring data privacy through covert communication, addressing the security needs of resource-constrained IoT devices. These use cases underscore the system’s versatility and practical relevance in addressing modern wireless communication challenges, particularly in secure and contested scenarios.

Moreover, ISCCO systems face not only the risk of communication and AirComp leakage but also the threat to the security of sensing information. Sensing data may be vulnerable to attacks from sensing eavesdroppers, who can silently intercept sensing results within ISAC systems without actively transmitting their signals^20^. Based on intercepted sensing information, attackers can infer the behavior of the associated physical systems and potentially initiate actions that degrade system performance. This passive eavesdropping on sensing information introduces additional privacy and security challenges, necessitating advanced mechanisms to ensure the confidentiality and integrity of sensing data^21–24^. In this context, hybrid radar fusion approaches proposed for ISAC systems^25^ have shown promise in enhancing sensing accuracy, which could serve as a foundation for improving robustness against sensing eavesdropping in STAR-RIS-assisted covert ISCCO systems. The study^26^ investigates the precoder design for single-ISAC transmitter scenarios based on sensor beam pattern distortion, and the authors of^27^ further consider precoder design in cell-free ISAC systems aimed at maximizing sensing signal-to-noise ratio (SNR). However, these studies focus on single communication systems or ISAC systems and are not applicable to covert ISCCO systems. The simultaneous consideration of communication, sensing, and AirComp concealment remains an area requiring further exploration.

In ISCCO systems, the integration of AirComp into communication systems undoubtedly increases the likelihood of eavesdropping^28,29^. At the same time, sensing data may be susceptible to attacks from sensing eavesdroppers^30^, who can deduce the behavior of the relevant physical systems based on intercepted sensing information, potentially leading to further actions that compromise system performance^31^. These unresolved issues have motivated our work. To address these challenges, this paper investigates a STAR-RIS-assisted ISCCO system that simultaneously supports covert sensing, communication, and computation. Specifically, multiple multi-antenna sensors transmit radar signals through STAR-RIS to a multi-antenna server for target detection and data symbol aggregation using Air-Comp. A dual-functional radar sensing and AirComp scheme is adopted, where the antennas at each sensor are divided into two groups: one for radar sensing and the other for data transmission. Given that the key performance indicators for radar sensing and AirComp are target estimation and MSE of function computation, respectively, there exists a natural trade-off between the performance of these two functions, as reflected in the beamforming design. This introduces a coupling between sensing and AirComp, requiring joint design of radar signal beamforming, data transmission beamforming, and data aggregation beamforming, while also adhering to concealment threshold constraints. The novelty and major contributions of this paper can be summarized as follows:

- We propose a novel optimization framework that directly incorporates these expressions as the objective (minimizing MSE for reliability) and constraints (ensuring covertness for security), enabling joint optimization of beamformers ($\textbf{W}_m$, $\textbf{F}_m$, $\textbf{A}$) and the STAR-RIS matrix ($\Theta$). The closed-form expressions simplify the formulation of the non-convex problem (P1), facilitating an efficient solution via a smoothed exact penalty algorithm with CCCP. Together, these contributions establish a unified approach to balance reliability and security in the covert ISCCO system.
- To ensure system reliability, we derive closed-form expressions for reliability (i.e., AirComp MSE) and security (i.e., covert communication rate $\mathscr {D}\left( \mathbb {P}_0 | \mathbb {P}_1\right) _C$ and covert sensing probability $\tilde{p}\left( \left\{ {{\textbf{F}}_{m}} \right\} ,\textbf{S} \right)$), providing analytical insights into the system’s performance under covert constraints.
- An optimization problem is formulated to minimize AirComp MSE by optimizing the data transmission beamformer, radar sensing beamformer, data aggregation beamformer, and STAR-RIS matrix. To solve this non-convex optimization problem, a smooth exact penalty algorithm with twice-continuous differentiability is proposed, transforming the optimization problem into a convex-concave form to seek a locally optimal solution.

## System and channel models

In this section, the system and channel models are described, and the related performance is derived and analyzed.

### A. System model

**Fig. 1:**
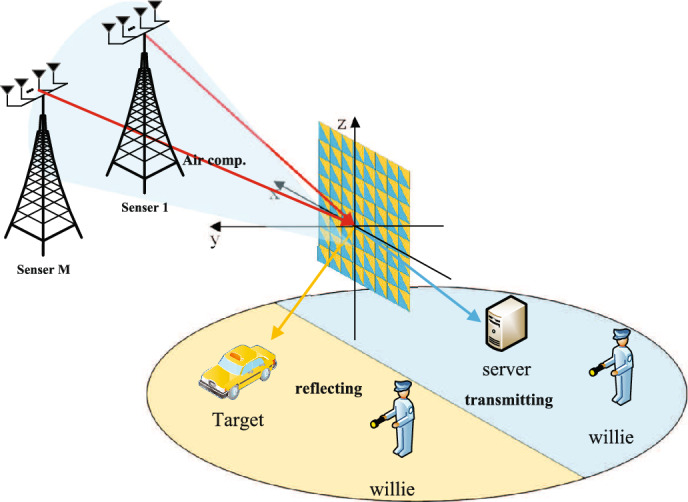
STAR-RIS assisted covert ISCCO system framework.

As illustrated in Figure 1, the sensors are designed to perform sensing tasks while providing communication and computation services. The radar senses a target and simultaneously transmits the received sensing data to a server equipped with *N* receiving antennas. The data is superimposed during transmission from the sensor’s communication transmit antenna to the STAR-RIS through the channel, enabling Air-Comp. The server then demodulates the necessary computation results. Each sensor has ${N_s}$ antennas divided into two groups, ${N_s} = {N_r} + {N_c}$,where antennas ${N_r}$ are used for radar sensing and ${N_c}$ antennas are used for data transmission. The sensor’s radar sensing antennas ${N_r}$ are further divided into two subgroups, ${N_r} = {N_{rt}} + {N_{rr}}$, with ${N_{rt}}$ antennas dedicated to transmitting radar sensing signals and ${N_{rr}}$ antennas for receiving echo signals reflected from the target.

Assuming that the direct links between the sensors and the sensing target, as well as between the sensors and the communication target server, are severely blocked and thus unusable, STAR-RIS is introduced into the system to enhance both communication and sensing. STAR-RIS is equipped with a uniform planar array (UPA) consisting of *N* passive transmission-reflection (T-R) elements, denoted as set $\mathscr {N}$. The entire space is divided by STAR-RIS into two half-spaces, with one half dedicated to sensing as the reflection region, and the other half designated for communication as the transmission region. The sensing region contains a sensing target and a sensing signal eavesdropper, while the communication space includes a server and a communication signal eavesdropper. The use of STAR-RIS also allows us to avoid interference between the sensing and communication signals. We consider a time block of length *T*, during which the communication channels and the parameters of the sensing target remain approximately constant. It is further assumed that perfect CSI for all channels is available at both the sensors and the STAR-RIS to explore the upper bound of covert performance.

This paper assumes perfect CSI at all nodes (including sensors, the server, and STAR-RIS) to simplify the analysis of the complex system model and explore performance upper bounds. Given that the ISCCO system integrates communication, sensing, and computation with multi-variable optimization (e.g., beamforming and STAR-RIS matrix), introducing imperfect CSI would significantly complicate the derivations and solution process. Thus, we focus on the perfect CSI scenario initially, with future work planned to investigate the effects of imperfect CSI. Additionally, for eavesdroppers, we assume they possess perfect CSI from themselves to legitimate users, representing the most stringent eavesdropping condition^13^. The optimization results under this assumption reflect the system’s performance in the worst-case scenario, providing a theoretical benchmark for optimal covert capability.

Therefore, the STAR-RIS operates in Energy Splitting (ES) mode to support simultaneous transmission and reflection. We define $\varvec{\Theta }_{t} \in \mathbb {C}^{N \times N}$ and $\varvec{\Theta }_{r} \in \mathbb {C}^{N \times N}$ as the transmission and reflection coefficients, respectively, which are expressed as follows:

1 $$\begin{aligned} {{\varvec{\Theta }}_i} = {\textrm{diag}}({\beta _{i,1}}{e^{j{\phi _{i,1}}}},...,{\beta _{i,N}}{e^{j{\phi _{i,N}}}}), \forall i \in \{ t,r\} \end{aligned}$$

where ${{\beta }_{i,n}}\in [0,1],\forall n\in \mathscr {N}$ represents the amplitude response of the *n*-th element, and ${{\varphi }_{i,n}}\in [0,2\pi ],\forall n\in \mathscr {N}$ represents the phase shift response of the *n*-th element. Specifically, the amplitude and phase shift responses are determined by the specific resistance and reactance of the elements in the STAR-RIS, with the amplitude responses required to satisfy the following energy conservation law:

2 $$\begin{aligned} \beta _{t,n}^{2}+\beta _{r,n}^{2}=1,\forall n\in {\mathscr {N}} \end{aligned}$$

where ${{\beta }_{t,n}}$ represents the amplitude response for the transmission surface, and ${{\beta }_{r,n}}$ represents the amplitude response for the reflection surface.

To reduce the manufacturing costs and minimize losses associated with STAR-RIS, the literature^32^ proposes a low-cost passive STAR-RIS, under which the phase shift response must satisfy the following condition:

3 $$\begin{aligned} \cos ({{\phi }_{t,n}}-{{\phi }_{t,n}})=0,\forall n\in {\mathscr {N}} \end{aligned}$$

### B. Practical feasibility considerations

While the proposed STAR-RIS-assisted covert ISCCO system demonstrates significant theoretical potential, its practical implementation involves several challenges that merit discussion.

Hardware and Deployment: The STAR-RIS relies on precise control of its transmission and reflection coefficients ($\Theta _t$ and $\Theta _r$), requiring advanced hardware capable of real-time phase and amplitude adjustments. Current RIS prototypes, primarily tested in sub-6 GHz bands, show promise, but scaling to mmWave frequencies, as considered in our simulations, demands further advancements in tunable metasurfaces with low power consumption^33^. Deploying STAR-RIS in dynamic environments, such as urban settings with moving obstacles, necessitates adaptive algorithms to track channel variations, potentially leveraging machine learning for efficient coefficient tuning.

Computational Complexity: The proposed optimization framework, based on a smoothed exact penalty algorithm with CCCP, involves iterative solutions for non-convex problems, which may be computationally intensive for real-time applications. In practice, simplified heuristic algorithms or hardware-accelerated implementations (e.g., using FPGA or GPU platforms) could reduce latency, making the system viable for time-sensitive scenarios like autonomous vehicle networks. Alternatively, pre-computed look-up tables for STAR-RIS configurations could trade off optimality for speed in static environments.

CSI Acquisition and Robustness: The assumption of perfect CSI at all nodes simplifies the analysis but may not hold in real-world scenarios due to channel estimation errors, feedback delays, or partial CSI availability. Robust optimization techniques, such as those based on worst-case CSI uncertainty models^34^, could mitigate these issues. Additionally, leveraging pilot-based channel estimation with reduced overhead or exploiting channel reciprocity in TDD systems could enhance feasibility.

Use Cases and Scalability: The integrated covert sensing, communication, and computation framework is well-suited for applications requiring high security and low latency, such as secure IoT networks for smart cities or collaborative computation in autonomous vehicle fleets. However, scaling the system to support a large number of sensors (*M*) or STAR-RIS elements (*N*) increases both hardware costs and optimization complexity. Hierarchical architectures, where multiple STAR-RIS units cooperate under a central controller, could address scalability, though this requires further investigation into synchronization and interference management.

These considerations highlight the trade-offs between theoretical performance and practical deployment. Ongoing advancements in RIS hardware, low-complexity algorithms, and robust CSI acquisition are expected to bridge this gap, paving the way for real-world implementation of the proposed ISCCO system.

### C. Signal model and air computation MSE

Let the data symbol transmitted by the *m*-th sensor during the *T*-th time slot be represented as the vector

4 $$\begin{aligned} {{\textbf{d}}_{m}}[t]={{\textbf{W}}_{m}}{{\textbf{x}}_{m}}[t]+{{\textbf{F}}_{m}}{{\textbf{s}}_{m}}[t] \end{aligned}$$

it is assumed that all communication signals participate in air computation. ${{\textbf{W}}_{m}}\in {{\mathbb {C}}^{{{N}_{c}}\times M}}$ represents the data transmission beamformer, and ${{\textbf{F}}_{m}}\in {{\mathbb {C}}^{{{N}_{rt}}\times M}}$ represents the radar sensing beamformer. Due to the limited transmission power of each sensor, the design of the beamformers must satisfy the following power constraints:

5 $$\begin{aligned} \operatorname {tr}\left( {{\textbf{W}}_{m}}{{\textbf{W}}_{m}}^{H} \right) +\operatorname {tr}\left( {{\textbf{F}}_{m}}{{\textbf{F}}_{m}}^{H} \right) \le P,\forall m \end{aligned}$$

Assuming that the communication data symbols across different sensors and functions are independently and identically distributed (i.i.d.), with zero mean and unit var-iance, they satisfy ${{\mathbb {E}}_{t}}\left[ {{\textbf{x}}_{m}}[t]\textbf{x}_{m}^{H}[t] \right] =\textbf{I}$ and ${{\mathbb {E}}_{t}}\left[ {{\textbf{x}}_{m}}[t]\textbf{x}_{i}^{H}[t] \right] =\textbf{0},\forall i\ne m$. The radar signals ${{\textbf{s}}_{m}}[t]$ are also assumed to be i.i.d. with zero mean. The data stream signals are statistically independent of the radar signals. We denote ${{\textbf{h}}_{RS}}\in {{\mathbb {C}}^{N}}$, ${{\textbf{h}}_{CS}}\in {{\mathbb {C}}^{N}}$, ${{\textbf{h}}_{SS}}\in {{\mathbb {C}}^{M\times N}}$, and ${{\textbf{h}}_{ST}}\in {{\mathbb {C}}^{M\times N}}$ as the channel gains from the radar antennas of the sensor to STAR-RIS, from the communication antennas of the sensor to STAR-RIS, from STAR-RIS to the server, and from STAR-RIS to the target, respectively. It is assumed that the channel conditions are flat and vary slowly within one transmission time slot. Furthermore, since various channel estimation schemes for STAR-RIS-assisted wireless transmission have been proposed^35,36^, we assume that perfect CSI is available for all channels except the eavesdropper channel ${{\textbf{h}}_{ST}}\in {{\mathbb {C}}^{M\times N}}$. In the next section, we will derive the target position estimation based on ISCCO (Integrated Sensing, Communication, and Computation Optimization). The target-reflected signal ${{\textbf{y}}_{m}}[t]\in { ^{{{N}_{rr}}\times 1}}$ received by the *m* -th sensor can be expressed as:

6 $$\begin{aligned} {{\textbf{y}}_{m}}[t]={{\textbf{G}}_{m}}{{\textbf{F}}_{m}}{{\textbf{s}}_{m}}[t]+{{\textbf{n}}_{r}}[t] \end{aligned}$$

where ${{\textbf{G}}_{m}}=\alpha ({{\textbf{h}}_{RS}}{{\varvec{\Theta }}_{r}}{{\textbf{h}}_{ST}}){{({{\textbf{h}}_{RS}}{{\varvec{\Theta }}_{r}}{{\textbf{h}}_{ST}})}^{H}}$ represents the composite channel parameter of the sensing signal transmitted by the *m* -th sensor, $\alpha$ represents the complex radar reflection gain of the target, and ${{\textbf{n}}_r} = {\mathop {\textrm{vec}}\nolimits } \left( {{{\textbf{N}}_r}} \right) \in {\mathscr {C}\mathscr {N}}\left( {{\textbf{0}},{{\textbf{R}}_{{n_r}}}} \right)$ is the Additive White Gaussian Noise. The signal received at the Access Point (AP) $\widehat{\textbf{z}}[t]\in { ^{M\times 1}}$ can be expressed as:

7 $$\begin{aligned} \widehat{\textbf{z}}[t]={{\textbf{A}}^{H}}\sum \limits _{m=1}^{M}{\left( {{\textbf{H}}_{m}}{{\textbf{W}}_{m}}{{\textbf{x}}_{m}}[t]+{{\textbf{R}}_{m}}{{\textbf{F}}_{m}}{{\textbf{s}}_{m}}[t] \right) }+{{\textbf{A}}^{H}}{{\textbf{n}}_{c}}[t] \end{aligned}$$

where ${{\textbf{H}}_{m}}={{\textbf{h}}_{CS}}{{\varvec{\Theta }}_{t}}{{\textbf{h}}_{SS}}\in {{\mathbb {C}}^{M\times {{N}_{c}}}}$ and ${{\textbf{R}}_{m}}={{\textbf{h}}_{RS}}{{\varvec{\Theta }}_{t}}{{\textbf{h}}_{SS}}\in {{\mathbb {C}}^{M\times {{N}_{rt}}}}$ represent the data symbol channel and radar signal channel between the AP and the *m*-th sensor, respectively. The AWGN (Additive White Gaussian Noise) vector ${{\textbf{n}}_{c}}\in { ^{M\times 1}}$ is statistically independent of ${{\textbf{x}}_{m}}[t]$ and ${{\textbf{s}}_{m}}[t]$. The MSE between the estimated function value and the true value can be expressed as:

8 $$\begin{aligned} \begin{array}{l} \mathbb {E}_{t}\left[ \left| \textbf{A}^{H} \sum _{m=1}^{M}\left( \textbf{H}_{m} \textbf{W}_{m} \textbf{x}_{m}[t]+\textbf{R}_{m} \textbf{F}_{m} \textbf{s}_{m}[t]\right) +\textbf{A}^{H} \textbf{n}_{c}[t]-\sum _{m=1}^{M} \textbf{x}_{m}[t]\right| ^{2}\right] \\ =\sum _{m=1}^{M} \operatorname {tr}\left( \left( \textbf{A}^{H} \textbf{H}_{m} \textbf{W}_{m}-\textbf{I}\right) \left( \textbf{A}^{H} \textbf{H}_{m} \textbf{W}_{m}-\textbf{I}\right) ^{H}\right) \\ +\sum _{m=1}^{M} \operatorname {tr}\left( \textbf{A}^{H} \textbf{R}_{m} \textbf{F}_{m} \textbf{F}_{m}^{H} \textbf{R}_{m}^{H} \textbf{A}\right) +\sigma _{c}^{2} \operatorname {tr}\left( \textbf{A A}^{H}\right) \end{array} \end{aligned}$$

### D. Target position estimation model

Since the channel between the BS (Base Station) and the STAR-RIS is known, we can configure the STAR-RIS to receive sensing signals, allowing the signal received at the sensor to be expressed as:

9 $$\begin{aligned} {{\textbf{Y}}_{r}}=\alpha \textbf{a}(\eta ,\phi ){{\textbf{b}}^{T}}(\eta ,\phi ){{\varvec{\Theta }}_{r}}{{\textbf{h}}_{RS}}{{\textbf{s}}_{m}}[t]+{{\textbf{N}}_{r}} \end{aligned}$$

where $\alpha$ is a complex constant that includes the round-trip path loss and the target Radar Cross Section (RCS), $\textbf{a}(\eta ,\phi )$ represents the steering vector of the sensing receiver elements, ${{\textbf{b}}^{T}}(\eta ,\phi )$ denotes the steering vector of the STARS reflection/transmission elements, ${{\varvec{\Theta }}_{r}}\in { ^{N\times N}}$ is the reflection coefficient, and ${{\textbf{N}}_{r}}$ is an additive white Gaussian noise (AWGN) component. Considering the Swerling-I model, during the transmission of the communication and sensing symbols *T* -th, it is assumed that the RCS fluctuates slowly and that the round-trip sensing channel remains constant.

The covariance matrix can be derived as:

10 $$\begin{aligned} {{\text {R}}_{\textbf{x}}}=\textbf{W}{{\textbf{W}}^{H}}\approx \frac{1}{T}\textbf{S}{{\textbf{S}}^{H}} \end{aligned}$$

By vectorizing ${{\textbf{Y}}_{r}}=\left[ {{\textbf{y}}_{r}}(1),\ldots ,{{\textbf{y}}_{r}}(T) \right]$, we obtain:

11 $$\begin{aligned} {{\textbf{y}}_{r}}=\operatorname {vec}\left( {{\textbf{Y}}_{r}} \right) =\textbf{p}+{{\textbf{n}}_{r}} \end{aligned}$$

where $$\textbf{p}=\operatorname {vec}\left( \alpha \textbf{a}(\eta ,\phi ){{\textbf{b}}^{T}}(\eta ,\phi )\Theta \textbf{HX} \right)$$, $${{\textbf{n}}_{r}}=\operatorname {vec}\left( {{\textbf{N}}_{r}} \right) \in {{CN}}\left( \textbf{0},{{\textbf{R}}_{{{n}_{r}}}} \right)$$ and $${{\textbf{R}}_{{{n}_{r}}}}=\sigma _{r}^{2}{{\textbf{I}}_{{{M}_{s}}T}}$$.

Define the target sensing estimation parameter as $\widetilde{\varvec{\xi }}={{\left[ {{\widetilde{\varvec{\varphi }}}^{T}},{{\widetilde{\varvec{\alpha }}}^{T}} \right] }^{T}}$, where $\widetilde{{\varphi }}={{[\eta ,\phi ]}^{T}}$ and $\widetilde{\varvec{\alpha }}={{[{\mathscr {R}}e(\alpha ),{\mathscr {I}}m(\alpha )]}^{T}}$ represent the relevant components. Consequently, the Fisher Information Matrix (FIM) can be derived as:

12 $$\begin{aligned} \mathbf {J=}\left[ \begin{matrix} {{\textbf{J}}_{\varvec{\tilde{\varphi }\tilde{\varphi }}}} & {{\textbf{J}}_{\varvec{\tilde{\varphi }\tilde{\alpha }}}} \\ \textbf{J}_{\varvec{\tilde{\varphi }\tilde{\alpha }}}^{T} & {{\textbf{J}}_{\varvec{\tilde{\alpha }\tilde{\alpha }}}} \\ \end{matrix} \right] \end{aligned}$$

The partial derivatives of $\textbf{p}$ with respect to $\widetilde{\varvec{\varphi }}$ and $\widetilde{\varvec{\alpha }}$ are given as fol-lows:

13 $$\begin{aligned} \frac{\partial \textbf{p}}{\partial \widetilde{\varvec{\varphi }}}=\left[ \alpha \operatorname {vec}\left( {{\widehat{\textbf{A}}}_{\eta }}\Theta \textbf{HX} \right) ,\alpha \operatorname {vec}\left( {{\widehat{\textbf{A}}}_{\phi }}\Theta \textbf{HX} \right) \right] \end{aligned}$$

14 $$\begin{aligned} \frac{\partial \textbf{p}}{\partial \widetilde{\varvec{\alpha }}}=[1,j]\otimes \operatorname {vec}(\textbf{A}\Theta \textbf{HX}) \end{aligned}$$

where $\textbf{A}=\textbf{a}(\eta ,\phi ){{\textbf{b}}^{T}}(\eta ,\phi )$ represents the relevant components. To compute the partial derivatives with respect to $\eta$ and $\phi$, we rewrite the steering vector as follows:

15 $$\begin{aligned} \textbf{a}(\eta ,\phi )=\frac{1}{\sqrt{{{M}_{s}}}}{{e}^{-j{{\textbf{v}}_{s}}}},\textbf{b}(\eta ,\phi )=\frac{1}{\sqrt{M}}{{e}^{-jv}} \end{aligned}$$

where ${{\textbf{v}}_{s}}=[2\pi /\lambda ]\left( {{\mathbf {\mu }}_{sY}}\sin \eta \sin \phi {{d}_{y}}+{{\mathbf {\mu }}_{sZ}}\cos \phi {{d}_{z}} \right)$, $\textbf{v}=[2\pi /\lambda ]\left( {{\mathbf {\mu }}_{Y}}\sin \eta \sin \phi {{d}_{y}}+{{\mathbf {\mu }}_{Z}}\cos \phi {{d}_{z}} \right)$. ${{\mathbf {\mu }}_{sY}}$ and ${{\mathbf {\mu }}_{sZ}}$ denote the indices of the elements corresponding to the sensing elements in the Y-axes and Z-axes, and ${{\mathbf {\mu }}_{Y}}$ and ${{\mathbf {\mu }}_{Z}}$ denote the indices of the elements corresponding to the reflection elements. Consequently, the partial derivatives of $\textbf{A}$ can be expressed as:

16 $$\begin{aligned} & \begin{array}{l} \widehat{\textbf{A}}_{\eta }=\frac{\partial \textbf{A}}{\partial \eta }=\frac{\partial \textbf{a}(\eta , \phi )}{\partial \eta } \textbf{b}^{T}(\eta , \phi )+\textbf{a}(\eta , \phi ) \frac{\partial \textbf{b}^{T}(\eta , \phi )}{\partial \eta } \\ =-j \frac{2 \pi }{\lambda \sqrt{M_{s} M}} \cos \eta \sin \phi d_{y} \times \left( \operatorname {diag}\left\{ \varvec{\mu }_{s Y}\right\} \textbf{a b}^{T}+\textbf{a b}^{T} \operatorname {diag}\left\{ \varvec{\mu }_{Y}\right\} \right) \end{array} \end{aligned}$$

17 $$\begin{aligned} & \begin{array}{l} \widehat{\textbf{A}}_{\phi }=\frac{\partial \textbf{A}}{\partial \phi }=\frac{\partial \textbf{a}(\eta , \phi )}{\partial \phi } \textbf{b}^{T}(\eta , \phi )+\textbf{a}(\eta , \phi ) \frac{\partial \textbf{b}^{T}(\eta , \phi )}{\partial \phi } \\ =-j \frac{2 \pi }{\lambda \sqrt{M_{s} M}} \sin \eta \cos \phi d_{y} \times \left( \operatorname {diag}\left\{ \varvec{\mu }_{s Y}\right\} \textbf{a b}^{T}+\textbf{a b}^{T} \operatorname {diag}\left\{ \varvec{\mu }_{Y}\right\} \right) \\ +j \frac{2 \pi }{\lambda \sqrt{M_{s} M}} \sin \phi d_{z} \times \left( \operatorname {diag}\left\{ \varvec{\mu }_{s Z}\right\} \textbf{a b}^{T}+\textbf{a b}^{T} \operatorname {diag}\left\{ \varvec{\mu }_{Z}\right\} \right) \end{array} \end{aligned}$$

Therefore, ${{\textbf{J}}_{\mathbf {\tilde{\varphi }\tilde{\varphi }}}}$ can be computed as:

18 $$\begin{aligned} & \begin{array}{l} {{\textbf{J}}_{\widetilde{\mathbf{\varphi }}\widetilde{\mathbf{\varphi }}}} = \frac{2}{{\sigma _r^2}}{\mathscr {R}}e\left\{ {\frac{{\partial {{\textbf{p}}^H}}}{{\partial \widetilde{\mathbf{\varphi }}}}\frac{{\partial {\textbf{p}}}}{{\partial \widetilde{\mathbf{\varphi }}}}} \right\} = \frac{2}{{\sigma _r^2}}{\mathscr {R}}e\left\{ \begin{array}{l} \left[ {\begin{array}{*{20}{c}} {{\alpha ^*}{\mathop {\textrm{vec}}\nolimits } {{\left( {{{\widehat{\textbf{A}}}_\eta }\Theta {\textbf{HX}}} \right) }^H}}\\ {{\alpha ^*}{\mathop {\textrm{vec}}\nolimits } {{\left( {{{\widehat{\textbf{A}}}_\phi }\Theta {\textbf{HX}}} \right) }^H}} \end{array}} \right] \\ \left[ {\alpha {\mathop {\textrm{vec}}\nolimits } \left( {{{\widehat{\textbf{A}}}_\eta }\Theta {\textbf{HX}}} \right) ,\alpha {\mathop {\textrm{vec}}\nolimits } \left( {{{\widehat{\textbf{A}}}_\phi }\Theta {\textbf{HX}}} \right) } \right] \end{array} \right\} \\ = \frac{{2|\alpha {|^2}}}{{\sigma _r^2}}{\mathscr {R}}e\left\{ {\left[ {\begin{array}{*{20}{c}} {{\mathop {\textrm{tr}}\nolimits } \left( {{{\widehat{\textbf{A}}}_\eta }\Theta {\textbf{HX}}{{\textbf{X}}^H}{{\textbf{H}}^H}{\Theta ^H}\widehat{\textbf{A}}_\eta ^H} \right) ,{\mathop {\textrm{tr}}\nolimits } \left( {{{\widehat{\textbf{A}}}_\phi }\Theta {\textbf{HX}}{{\textbf{X}}^H}{{\textbf{H}}^H}{\Theta ^H}\widehat{\textbf{A}}_\eta ^H} \right) }\\ {{\mathop {\textrm{tr}}\nolimits } \left( {{{\widehat{\textbf{A}}}_\eta }\Theta {\textbf{HX}}{{\textbf{X}}^H}{{\textbf{H}}^H}{\Theta ^H}\widehat{\textbf{A}}_\phi ^H} \right) ,{\mathop {\textrm{tr}}\nolimits } \left( {{{\widehat{\textbf{A}}}_\phi }\Theta {\textbf{HX}}{{\textbf{X}}^H}{{\textbf{H}}^H}{\Theta ^H}\widehat{\textbf{A}}_\phi ^H} \right) } \end{array}} \right] } \right\} \\ = \frac{{2|\alpha {|^2}T}}{{\sigma _r^2}}{\mathscr {R}}e\left\{ {\left[ {\begin{array}{*{20}{l}} {{\mathop {\textrm{tr}}\nolimits } \left( {{{\widehat{\textbf{A}}}_\eta }\Theta {\textbf{HRx}}{{\textbf{H}}^H}{\Theta ^H}\widehat{\textbf{A}}_\eta ^H} \right) ,{\mathop {\textrm{tr}}\nolimits } \left( {{{\widehat{\textbf{A}}}_\phi }\Theta {\textbf{H}}{{\textbf{R}}_{\textbf{x}}}{{\textbf{H}}^H}{\Theta ^H}\widehat{\textbf{A}}_\eta ^H} \right) }\\ {{\mathop {\textrm{tr}}\nolimits } \left( {{{\widehat{\textbf{A}}}_\eta }\Theta {\textbf{HR}}{{\textbf{R}}_{\textbf{x}}}{{\textbf{H}}^H}{\Theta ^H}\widehat{\textbf{A}}_\phi ^H} \right) ,{\mathop {\textrm{tr}}\nolimits } \left( {{{\widehat{\textbf{A}}}_\phi }\Theta {\textbf{H}}{{\textbf{R}}_{\textbf{x}}}{{\textbf{H}}^H}{\Theta ^H}\widehat{\textbf{A}}_\phi ^H} \right) } \end{array}} \right] } \right\} \end{array} \end{aligned}$$

The other fractions can be similarly derived as:

19 $$\begin{aligned} & \begin{array}{l} {{\textbf{J}}_{\tilde{\varphi }\tilde{\alpha }}} = \frac{2}{{\sigma _r^2}}{\mathscr {R}}e\left\{ {\left[ {\begin{array}{*{20}{l}} {{\alpha ^*}{\mathop {\textrm{vec}}\nolimits } {{\left( {{{\widehat{\textbf{A}}}_\eta }\Theta {\textbf{HX}}} \right) }^H}}\\ {{\alpha ^*}{\mathop {\textrm{vec}}\nolimits } {{\left( {{{\widehat{\textbf{A}}}_\phi }\Theta {\textbf{HX}}} \right) }^H}} \end{array}} \right] ([1,j] \otimes {\mathop {\textrm{vec}}\nolimits } ({\textbf{A}}\Theta {\textbf{HX}}))} \right\} \\ = \frac{2}{{\sigma _r^2}}{\mathscr {R}}e\left\{ {\left[ {\begin{array}{*{20}{l}} {{\alpha ^*}{\mathop {\textrm{tr}}\nolimits } \left( {{\textbf{A}}\Theta {\textbf{HX}}{{\textbf{X}}^H}{{\textbf{H}}^H}{\Theta ^H}\widehat{\textbf{A}}_\eta ^H} \right) }\\ {{\alpha ^*}{\mathop {\textrm{tr}}\nolimits } \left( {{\textbf{A}}\Theta {\textbf{HX}}{{\textbf{X}}^H}{{\textbf{H}}^H}{\Theta ^H}\widehat{\textbf{A}}_\phi ^H} \right) } \end{array}} \right] \otimes [1,j]} \right\} \\ = \frac{{2T}}{{\sigma _r^2}}{\mathscr {R}}e\left\{ {\left[ {\begin{array}{*{20}{c}} {{\alpha ^*}{\mathop {\textrm{tr}}\nolimits } \left( {{\textbf{A}}\Theta {\textbf{HR}}{{\textbf{R}}_{\textbf{x}}}{{\textbf{H}}^H}{\Theta ^H}\widehat{\textbf{A}}_\eta ^H} \right) }\\ {{\alpha ^*}{\mathop {\textrm{tr}}\nolimits } \left( {{\textbf{A}}\Theta {\textbf{HR}}{{\textbf{R}}_{\textbf{x}}}{{\textbf{H}}^H}{\Theta ^H}\widehat{\textbf{A}}_\phi ^H} \right) } \end{array}} \right] \otimes [1,j]} \right\} \end{array} \end{aligned}$$

20 $$\begin{aligned} & \begin{array}{l} {{\textbf{J}}_{\tilde{\alpha }\tilde{\alpha }}} = \frac{2}{{\sigma _r^2}}{\mathscr {R}}e\left\{ {{{([1,j] \otimes {\mathop {\textrm{vec}}\nolimits } ({\textbf{A}}\Theta {\textbf{HX}}))}^H}([1,j] \otimes {\mathop {\textrm{vec}}\nolimits } ({\textbf{A}}\Theta {\textbf{HX}}))} \right\} \\ = \frac{2}{{\sigma _r^2}}{\mathscr {R}}e\left\{ {{{[1,j]}^H}[1,j]{\mathop {\textrm{tr}}\nolimits } \left( {{\textbf{A}}\Theta {\textbf{HX}}{{\textbf{X}}^H}{{\textbf{H}}^H}{\Theta ^H}{{\textbf{A}}^H}} \right) } \right\} \\ = \frac{{2T}}{{\sigma _r^2}}{\mathscr {R}}e\left\{ {{{[1,j]}^H}[1,j]{\mathop {\textrm{tr}}\nolimits } \left( {{\textbf{A}}\Theta {\textbf{H}}{{\textbf{R}}_{\textbf{x}}}{{\textbf{H}}^H}{\Theta ^H}{{\textbf{A}}^H}} \right) } \right\} \end{array} \end{aligned}$$

Thus, each element in $\textbf{J}$ can be represented by the following equation:

21 $$\begin{aligned} {{\textbf{J}}_{i,j}} = {\mathop {\textrm{tr}}\nolimits } \left( {{\textbf{R}}_{{n_r}}^{ - 1}\frac{{\partial {{\textbf{R}}_{{n_r}}}}}{{\partial {{\widetilde{\mathbf{\xi }}}_i}}}{\textbf{R}}_{{n_r}}^{ - 1}\frac{{\partial {{\textbf{R}}_{{n_r}}}}}{{\partial {{\tilde{\xi }}_j}}}} \right) + 2{\mathscr {R}}e\left\{ {\frac{{\partial {{\textbf{p}}^H}}}{{\partial {{\widetilde{\mathbf{\xi }}}_i}}}{\textbf{R}}_{{n_r}}^{ - 1}\frac{{\partial {\textbf{p}}}}{{\partial {{\tilde{\xi }}_j}}}} \right\} \mathop = \limits ^{(a)} \frac{2}{{\sigma _r^2}}{\mathscr {R}}e\left\{ {\frac{{\partial {{\textbf{p}}^H}}}{{\partial {{\tilde{\xi }}_i}}}\frac{{\partial {\textbf{p}}}}{{\partial {{\tilde{\xi }}_j}}}} \right\} \end{aligned}$$

The CRB can then be obtained as:

22 $$\begin{aligned} {\mathop {\textrm{CRB}}\nolimits } (\tilde{\varphi }) = {\left[ {{{\textbf{J}}_{\tilde{\varphi }\tilde{\varphi }}} - {{\textbf{J}}_{\tilde{\varphi }\tilde{\alpha }}}{\textbf{J}}_{\tilde{\alpha }\tilde{\alpha }}^{ - 1}{\textbf{J}}_{\widetilde{\mathbf{\mu }}\tilde{\alpha }}^T} \right] ^{ - 1}} \end{aligned}$$

Subsequently, the closed-form expression of the CRB can be derived. Minimizing the CRB is achieved by optimizing the sensing waveform to match the predicted target direction, which requires the target’s estimated angle. Assuming the reasonable hypothesis that the target moves slowly, the target direction does not change significantly between adjacent coherent intervals. Therefore, the predicted angle is sufficient for waveform optimization^37^. This is a typical scenario in radar tracking, where prior knowledge of the target direction is used for system design.

## Warder probability models for communication, sensing and airComp

### A. Communication eavesdropping probability

AirComp can be considered to be carried out simultaneously with communication^29^. Therefore, the covert probability of AirComp and the covert probability of communication can be unified together for calculation. Next, we introduce the eaves-dropper model for communication. Since AirComp can be regarded as a form of Non-Orthogonal Multiple Access (NOMA), we assume that perfect CSI is available to Willie. Specifically, the channel gain between the STAR-RIS and Willie is denoted as ${{h}_{S{{W}_{C}}}}$, where Willie is equipped with a single antenna. This allows us to describe the fundamental performance gain achievable by STAR-RIS in a covert communication environment. Willie attempts to detect the presence of the transmission by distinguishing between the following hypotheses:

23 $$\begin{aligned} \left\{ {\begin{array}{*{20}{l}} {{{\widetilde{\mathscr {H}}}_0}:{y_w}[k] = {n_w}[k],}\\ {{{\widetilde{\mathscr {H}}}_1}:{y_w}[k] = {{\textbf{H}}_{m,{W_C}}}{{\textbf{W}}_m}{x_m}[t] + {n_w}[k]} \end{array}} \right. \end{aligned}$$

where ${{y}_{w}}[k]$ represents the signal received by Willie over the *k*-th channel, ${{\widetilde{{\mathscr {H}}}}_{0}}$ denotes the null hypothesis in which the device is not transmitting any information, ${{\widetilde{{\mathscr {H}}}}_{1}}$ represents the alternative hypothesis in which the device at the BS transmits information to the server, ${{p}_{i}}$ is the transmit power of the *i*-th sensor, and ${{n}_{w}}$ is the Additive Gaussian White Noise (AGWN) with zero mean and variance $\delta _{w}^{2}$. Additionally, ${{\textbf{H}}_{m,{{W}_{C}}}}={{\textbf{h}}_{CS}}{{\varvec{\Theta }}_{t}}{{\textbf{h}}_{S{{W}_{C}}}}$ represents the composite channel from the communication base station to the eavesdropper via the STAR-RIS. From this, we can derive the false alarm rate $Pr\left\{ {{{\mathscr {D}}}_{1}}|{{{\mathscr {H}}}_{0}} \right\}$ and the miss detection rate $Pr\left\{ {{{\mathscr {D}}}_{0}}|{{{\mathscr {H}}}_{1}} \right\}$ at Willie’s location, where ${{{\mathscr {D}}}_{1}}$ and ${{{\mathscr {D}}}_{0}}$ indicate the events of whether the device is transmitting or not. Therefore, the probability of detection error at Willie can be computed as:

24 $$\begin{aligned} \xi = \Pr \left\{ {{{\mathscr {D}}_1}\mid {{\mathscr {H}}_0}} \right\} + \Pr \left\{ {{{\mathscr {D}}_0}\mid {{\mathscr {H}}_1}} \right\} \end{aligned}$$

Willie’s objective is to minimize the detection error probability $\xi$ to identify the presence of OAC transmissions. The optimal criterion $\xi$ is the likelihood ratio test, which can be expressed as:

25 $$\begin{aligned} {\mathbb {P}_1}= & \prod \limits _{k = 1}^L f \left( {{y_w}[k]\mid {{\mathscr {H}}_1}} \right) \ge 1,\forall k \in {{\mathscr {D}}_1} \end{aligned}$$

26 $$\begin{aligned} {\mathbb {P}_0}= & \prod \limits _{k = 1}^L f \left( {{y_w}[k]\mid {{\mathscr {H}}_0}} \right) \le 1,\forall k \in {{\mathscr {D}}_0} \end{aligned}$$

where ${\mathbb {P}_{0}}$ and ${\mathbb {P}_{1}}$ represent the likelihood detections at Willie when the wireless channel uses ${{{\mathscr {H}}}_{0}}$ and ${{{\mathscr {H}}}_{1}}$, respectively. Using $f\left( {{y}_{w}}[k]\left| {{{\mathscr {H}}}_{0}} \right. \right) ={\mathscr {C}\mathscr {N}}\left( 0,\delta _{w}^{2} \right)$ and $f\left( {{y}_{w}}[k]\left| {{{\mathscr {H}}}_{1}} \right. \right) ={\mathscr {C}\mathscr {N}}\left( 0,{{(p_{m}^{C})}^{2}}{{\left| {{\textbf{H}}_{m,{{W}_{C}}}} \right| }^{2}}+\delta _{w}^{2} \right)$, we denote the likelihood detections under hypotheses ${{{\mathscr {H}}}_{0}}$ and ${{{\mathscr {H}}}_{1}}$ and criterion ${{\textbf{y}}_{w}}$. Under hypothesis ${{{\mathscr {H}}}_{0}}$, the received signal at Willie consists solely of AWGN, and the likelihood function of ${{\textbf{y}}_{w}}$ can be expressed as:

27 $$\begin{aligned} f\left( {{{\textbf{y}}_w}\left| {{{\mathscr {H}}_0}} \right. } \right) = \prod \limits _{k = 1}^L f \left( {{y_w}[k]\left| {{{\mathscr {H}}_0}} \right. } \right) = \frac{1}{{{{\left( {\pi \delta _w^2} \right) }^L}}}\exp \left( { - \frac{{\sum \limits _{k = 1}^L {{{\left| {{y_w}[k]} \right| }^2}} }}{{\delta _w^2}}} \right) \end{aligned}$$

Under hypothesis ${{{\mathscr {H}}}_{1}}$, the likelihood function of ${{\textbf{y}}_{w}}$ can be expressed as:

28 $$\begin{aligned} \begin{array}{l} f\left( {{{\textbf{y}}_w}\left| {{{\mathscr {H}}_1}} \right. } \right) = \prod \limits _{k = 1}^L f \left( {{y_w}[k]\left| {{{\mathscr {H}}_1}} \right. } \right) \\ = \frac{1}{{{{\left( {\pi \left( {{{(p_m^C)}^2}{{\left| {{{\textbf{H}}_{m,{W_C}}}} \right| }^2} + \delta _w^2} \right) } \right) }^L}}} \times \exp \left( { - \frac{{\sum \limits _{k = 1}^L {{{\left| {{y_w}[k]} \right| }^2}} }}{{{{(p_m^C)}^2}{{\left| {{{\textbf{H}}_{m,{W_C}}}} \right| }^2} + \delta _w^2}}} \right) \end{array} \end{aligned}$$

We can derive the optimal detection threshold ${{\xi }^{*}}$ at Willie’s location from Equation (24). However, the formal expression of ${{\xi }^{*}}\ge 1-{\epsilon }$ involves the Gamma function, making the optimization and solution process challenging. To address this issue, we employ the lower bound ${{\xi }^{*}}$^38^.

29 $$\begin{aligned} {\xi ^*} \ge 1 - \sqrt{\frac{1}{2}{\mathscr {D}}\left( {{\mathbb {P}_0}{\mathbb {P}_1}} \right) } \end{aligned}$$

here, ${\mathscr {D}}\left( {\mathbb {P}_{0}}\Vert {\mathbb {P}_{1}} \right)$ represents the Kullback-Leibler (KL) divergence between the hypotheses ${\mathbb {P}_{0}}$ and ${\mathbb {P}_{1}}$, derived from $f\left( {{y}_{w}}\left| {{H}_{0}} \right. \right)$ to $f\left( {{y}_{w}}\left| {{H}_{1}} \right. \right)$, based on the total variation distance bound on hypothesis testing performance and Pinsker’s inequality^39^.

30 $$\begin{aligned} {\mathscr {D}}{\left( {{\mathbb {P}_0}{\mathbb {P}_1}} \right) _C} = M\left[ {\ln \left( {\frac{{{{(p_m^C)}^2}{{\left| {{{\textbf{H}}_{m,{W_C}}}} \right| }^2} + \sigma _w^2}}{{\sigma _w^2}}} \right) } \right. \left. { + \frac{{\sigma _w^2}}{{{{(p_m^C)}^2}{{\left| {{{\textbf{H}}_{m,{W_C}}}} \right| }^2} + \sigma _w^2}} - 1} \right] \end{aligned}$$

In this work, we formally define a transmission (or sensing) as covert if it remains undetectable to an adversary within a specified detection probability threshold. Specifically, we adopt a stricter covertness constraint based on the KL divergence between the distributions under the null hypothesis ${{\mathbb {P}}_{0}}$ (no transmission) and the alternative hypothesis ${{\mathbb {P}}_{1}}$ (transmission occurs), given by ${\mathscr {D}}\left( {{\mathbb {P}}_{0}}\left\| {{\mathbb {P}}_{1}} \right. \right) \le 2{{\varepsilon }^{2}}$, where $\varepsilon \in \left( 0,0.5 \right)$ is a small, application-specific covertness threshold that bounds the adversary’s ability to reliably distinguish between the two hypotheses. This constraint guarantees that the sum of the false alarm and missed detection probabilities remains close to one, thereby ensuring low detectability. Compared to the more common constraint ${{\xi }^{*}}\ge 1-\varepsilon$, where is the minimum total detection error probability (i.e., the sum of type-I and type-II errors), the KL-divergence-based formulation offers a tighter and information-theoretic guarantee on covertness. This stricter constraint is particularly suitable for highly sensitive applications such as military or privacy-critical systems.

### B. Sensing eavesdropping probability

The sensing eavesdropping probability follows a similar principle. Since the transmitted signal ${{\textbf{s}}_{m}}[t]$ is assumed to be confidential, the sensing eaves-dropper lacks knowledge of ${{\textbf{s}}_{m}}[t]$. Consequently, the sensing eaves-dropper can only determine the presence of the target through energy detection. Let the beamforming vector used by the sensing eavesdropper be denoted as $\textbf{a}_{w}^{H}\left( \omega \right)$, where $\omega$ represents the Angle of Departure (AoD) from the target to the sensing eavesdropper. The signal received by the sensing eavesdropper can then be expressed as:

31 $$\begin{aligned} \tilde{r}(t)=\textbf{a}_{w}^{H}\left( \omega \right) \left( \sum \limits _{m=1}^{M}{\eta }{{\textbf{a}}_{w}}\left( \omega \right) \textbf{H}_{m,{{W}_{S}}}^{H}{{\textbf{s}}_{m}}[t]+\underbrace{\sum \limits _{m=1}^{M}{\textbf{U}_{m}^{H}}{{\textbf{s}}_{m}}[t]}_{\text {clutters}}+\widehat{\textbf{n}}\left( t \right) \right) \end{aligned}$$

where $\textbf{H}_{m,{{W}_{S}}}^{H}={{\textbf{h}}_{CS}}{{\varvec{\Theta }}_{r}}{{\textbf{h}}_{S{{W}_{S}}}}$ denotes the composite channel matrix from the radar to the eaves-dropper via the STAR-RIS, $\textbf{U}_{m}^{H}\in {\mathbb {C}^{N\times N}}$ rep-resents the composite clutter channel from the radar to the eavesdropper, and $\widehat{\textbf{n}}_q(t) \sim \mathscr{C}\mathscr{N}\left( \textbf{0}, \sigma _s^2 \textbf{I} \right)$ characterizes the Gaussian white noise. For convenience, we rewrite this as:

32 $$\begin{aligned} {\tilde{r}}(t) = {\textbf{H}}_{{W_S}}^H{\textbf{s}}[t] + {{\textbf{u}}^H}{\textbf{s}}[t] + {\textbf{a}}_w^H\left( \omega \right) \widehat{\textbf{n}}(t) \end{aligned}$$

where $\textbf{H}_{{{W}_{S}}}^{H}={{\left[ \eta \textbf{H}_{1,{{W}_{S}}}^{H},\ldots ,\eta \textbf{H}_{M,{{W}_{S}}}^{H} \right] }^{H}}$ is the equivalent composite channel matrix, ${{\textbf{u}}_{m}}={{\left[ \textbf{a}_{w}^{H}\left( \omega \right) \textbf{U}_{1}^{H},\ldots ,\textbf{a}_{w}^{H}\left( \omega \right) \textbf{U}_{M,n}^{H} \right] }^{H}}$ is the equivalent clutter channel, and $\hat{n}(t)=\textbf{a}_{w}^{H}\left( \omega \right) \widehat{\textbf{n}}(t)\sim {\mathscr {C}\mathscr {N}}\left( 0,N\sigma _{s}^{2} \right)$ represents the equivalent noise.

Let the null hypothesis ${{{\tilde{\mathscr {H}}}}_{0}}$ indicate the absence of a target within the sensing region, and the alternative hypothesis ${{{\tilde{\mathscr {H}}}}_{1}}$ represent the presence of a target. Accordingly, the hypotheses are formulated as:

33 $$\begin{aligned} \left\{ {\begin{array}{*{20}{l}} {{{\widetilde{\mathscr {H}}}_0}:{\tilde{r}}(t) = {{\textbf{u}}^H}{\textbf{s}}(t) + {\hat{n}}(t)}\\ {{{\widetilde{\mathscr {H}}}_1}:{{{\tilde{r}}}_q}(t) = {\textbf{H}}_{{W_S}}^H{\textbf{s}}(t) + {{\textbf{u}}^H}{\textbf{s}}(t) + {\hat{n}}(t)} \end{array}} \right. \end{aligned}$$

Next, we consider the eavesdropping probability at the sensing eavesdropper. Since the transmitted signal ${{\textbf{s}}_{m}}[t]$ is assumed to be confidential, the sensing eavesdropper lacks knowledge of ${{\textbf{s}}_{m}}[t]$. Consequently, the sensing eavesdropper can only determine the presence of a target through energy detection, which is provided by a detector ${{\left| \tilde{r}(t) \right| }^{2}}$^40^. Therefore, under hypotheses ${{{\tilde{\mathscr {H}}}}_{0}}$ and ${{{\tilde{\mathscr {H}}}}_{1}}$, the likelihood functions of the signal are respectively given by:

34 $$\begin{aligned} {p_0}\left( {{\tilde{r}}(t)} \right)= & \frac{1}{{\pi \zeta \left( {\left\{ {{{\textbf{F}}_m}} \right\} ,{\textbf{S}}} \right) }}\exp \left( { - \frac{{{{\left| {{\tilde{r}}(t)} \right| }^2}}}{{\zeta \left( {\left\{ {{{\textbf{F}}_m}} \right\} ,{\textbf{S}}} \right) }}} \right) \end{aligned}$$

35 $$\begin{aligned} {p_1}\left( {{\tilde{r}}(t)} \right)= & \frac{1}{{\pi \beta \left( {\left\{ {{{\textbf{F}}_m}} \right\} ,{\textbf{S}}} \right) }}\exp \left( { - \frac{{{{\left| {{\tilde{r}}(t)} \right| }^2}}}{{\beta \left( {\left\{ {{{\textbf{F}}_m}} \right\} ,{\textbf{S}}} \right) }}} \right) \end{aligned}$$

where $\zeta (\left\{ {{\textbf{w}}_{k}} \right\} ,\textbf{S})={{u}^{H}}\textbf{Ru}+N{{\sigma }^{2}}$, $\beta (\left\{ {{\textbf{F}}_{m}} \right\} ,\textbf{S})={{\left( \textbf{H}_{{{W}_{S}}}^{H}+\textbf{u} \right) }^{H}}\textbf{R}\left( \textbf{H}_{{{W}_{S}}}^{H}+\textbf{u} \right) +N{{\sigma }^{2}}$, $\textbf{R}=\sum \limits _{m=1}^{M}{{{\textbf{F}}_{m}}\textbf{F}_{m}^{H}}$. Based on the Neyman-Pearson criterion, the optimal detection rule for the sensing eavesdropper is given by:

36 $$\begin{aligned} {L_{\textrm{G}}}\left( {{\tilde{r}}(t)} \right) = \frac{{{p_0}\left( {{\tilde{r}}(t)} \right) }}{{{p_1}\left( {{\tilde{r}}(t)} \right) }}\gtreqless 1 \end{aligned}$$

Substituting Equations (34) and (35) into Equation (36), it can be reformulated as:

37 $$\begin{aligned} {\left| {{\tilde{r}}(t)} \right| ^2}\gtreqless \frac{{\zeta \left( {\left\{ {{{\textbf{F}}_m}} \right\} ,{\textbf{S}}} \right) \beta \left( {\left\{ {{{\textbf{F}}_m}} \right\} ,{\textbf{S}}} \right) }}{{\beta \left( {\left\{ {{{\textbf{F}}_m}} \right\} ,{\textbf{S}}} \right) - \zeta \left( {\left\{ {{{\textbf{F}}_m}} \right\} ,{\textbf{S}}} \right) }}\ln \left( {\frac{{\beta \left( {\left\{ {{{\textbf{F}}_m}} \right\} ,{\textbf{S}}} \right) }}{{\zeta \left( {\left\{ {{{\textbf{F}}_m}} \right\} ,{\textbf{S}}} \right) }}} \right) \end{aligned}$$

From Equations (34) and (35), the cumulative distribution functions (CDFs) under hypotheses $\textbf{T}$ and $\textbf{Y}$ are respectively given by:

38 $$\begin{aligned} \Pr \left( {{{\left| {{\tilde{r}}(t)} \right| }^2}\mid {{\widetilde{\mathscr {H}}}_0}} \right)= & 1 - \exp \left( { - \frac{{{{\left| {{\tilde{r}}(t)} \right| }^2}}}{{\zeta \left( {\left\{ {{{\textbf{F}}_m}} \right\} ,{\textbf{S}}} \right) }}} \right) \end{aligned}$$

39 $$\begin{aligned} \Pr \left( {{{\left| {{\tilde{r}}(t)} \right| }^2}\mid {{\widetilde{\mathscr {H}}}_1}} \right)= & 1 - \exp \left( { - \frac{{{{\left| {{\tilde{r}}(t)} \right| }^2}}}{{\beta \left( {\left\{ {{{\textbf{F}}_m}} \right\} ,{\textbf{S}}} \right) }}} \right) \end{aligned}$$

Substituting Equation (37) into Equation (39), we obtain the sensing eavesdropping probability as:

40 $$\begin{aligned} {\tilde{p}}\left( {\left\{ {{{\textbf{F}}_m}} \right\} ,{\textbf{S}}} \right) = {\left( {\frac{{\beta \left( {\left\{ {{{\textbf{F}}_m}} \right\} ,{\textbf{S}}} \right) }}{{\zeta \left( {\left\{ {{{\textbf{F}}_m}} \right\} ,{\textbf{S}}} \right) }}} \right) ^{ - \frac{{\zeta \left( {\left\{ {{{\textbf{F}}_m}} \right\} ,{\textbf{S}}} \right) }}{{\beta \left( {\left\{ {{{\textbf{F}}_m}} \right\} ,{\textbf{S}}} \right) - \zeta \left( {\left\{ {{{\textbf{F}}_m}} \right\} ,{\textbf{S}}} \right) }}}} \end{aligned}$$

## Problem formulation and optimization

In this section, to evaluate the MSE of AirComp, we consider the joint optimization of the data transmission beamformer ${{\textbf{W}}_{m}}$, radar sensing beamformer ${{\textbf{F}}_{m}}$, data aggregation beamformer $\textbf{A}$, and the STAR-RIS matrix. Specifically, we aim to optimize the MSE from Equation (8) under the amplitude and phase constraints from Equations (2) and (3), the power constraints from Equation (5), the sensing quality constraints from Equation (22), and the covert communication constraints from Equations (30) and (40).

The optimization framework builds upon the closed-form expressions derived for reliability and security. Specifically, the AirComp MSE, given by $\mathbb {E}\left[ \left| \textbf{s} - \hat{\textbf{s}}\right| ^2\right]$, quantifies computational reliability and serves as the objective function to be minimized. The security metrics, namely the covert communication rate $\mathscr {D}\left( \mathbb {P}_0 | \mathbb {P}_1\right) _C \le 2\varepsilon _C^2$ and covert sensing probability $\tilde{p}\left( \left\{ {{\textbf{F}}_{m}} \right\} ,\textbf{S} \right) \le 2\varepsilon _{S}^{2}$, are derived in closed form (e.g., using the Lambert $\mathscr {W}$ function for communication covertness), providing explicit constraints that limit signal detectability by eavesdroppers. These expressions are directly integrated into problem (P1), where the MSE defines the optimization goal, and the covertness constraints shape the feasible region for variables $\textbf{W}_m$, $\textbf{F}_m$, $\textbf{A}$, and $\Theta$. By leveraging these analytical results, we transform the complex joint design into a structured optimization problem, solvable through iterative techniques, thus ensuring that reliability and security are jointly optimized rather than treated in isolation.

41 $$\begin{aligned}&{\mathbf{(P1)}}\mathop {\min }\limits _{{\varvec{\Theta }},{\textbf{A}},\left\{ {{{\textbf{W}}_m}} \right\} ,\left\{ {{{\textbf{F}}_m}} \right\} } \sum \limits _{m = 1}^M {{\mathop {\textrm{tr}}\nolimits } } \left( {\left( {{{\textbf{A}}^H}{{\textbf{H}}_m}{{\textbf{W}}_m} - {\textbf{I}}} \right) {{\left( {{{\textbf{A}}^H}{{\textbf{H}}_m}{{\textbf{W}}_m} - {\textbf{I}}} \right) }^H}} \right) \\&+ \sum \limits _{m = 1}^M {{\mathop {{\textrm{tr}}}\nolimits } } \left( {{{\textbf{A}}^H}{{\textbf{R}}_m}{{\textbf{F}}_m}{\textbf{F}}_m^H{\textbf{R}}_m^H{\textbf{A}}} \right) + \sigma _c^2{\mathop {{\textrm{tr}}}\nolimits } \left( {{\textbf{A}}{{\textbf{A}}^H}} \right) \end{aligned}$$

41a $$\begin{aligned}&s.t.\quad {\mathop {{\textrm{tr}}}\nolimits } \left( {{{\left( {{{\textbf{F}}_m}{\textbf{F}}_m^H} \right) }^{ - 1}}} \right) \le \frac{{T{\eta _m}}}{{{N_{rr}}\sigma _r^2}},\forall m, \end{aligned}$$

41b $$\begin{aligned}&{\mathop {{\textrm{tr}}}\nolimits } \left( {{{\textbf{W}}_m}{{\textbf{W}}_m}^H} \right) + {\mathop {\textrm{tr}}\nolimits } \left( {{{\textbf{F}}_m}{{\textbf{F}}_m}^H} \right) \le P,\forall m., \end{aligned}$$

41c $$\begin{aligned}&{\mathop {\textrm{CRB}}\nolimits } (\tilde{\varphi }) \le \Upsilon , \end{aligned}$$

41d $$\begin{aligned}&\mathscr {D}\left( \mathbb {P}_{0} \Vert \mathbb {P}_{1}\right) _{C} \le 2 \epsilon _{C}^{2}, \end{aligned}$$

41e $$\begin{aligned}&\tilde{p}\left( \left\{ \textbf{F}_{m}\right\} , \textbf{S}\right) \le 2 \epsilon _{S} ^{2}. \end{aligned}$$

The optimization problem (P1) involves multiple constraints, including the CRB threshold $\operatorname {CRB}(\hat{\varphi }) \le \Upsilon$, covert communication constraint $\mathscr {D}\left( \mathbb {P}_0 | \mathbb {P}_1\right) _C \le 2\varepsilon _C^2$, and covert sensing constraint $\tilde{p}\left( \left\{ \textbf{F}_{m}\right\} , \textbf{S}\right) \le 2 \epsilon _{S} ^{2}$. These constraints are coupled through the beamforming vectors $\textbf{W}_m$, $\textbf{F}_m$, $\textbf{A}$, and the STAR-RIS matrix $\Theta$. For instance, a stringent CRB threshold requires higher power allocation to $\textbf{F}_m$ to enhance sensing SNR, which may increase the sensing eavesdropping probability $\tilde{p}\left( \left\{ \textbf{F}_{m}\right\} , \textbf{S}\right)$, potentially violating the covert sensing constraint. Similarly, the covert communication constraint limits the power and direction of $\textbf{W}_m$, which could degrade the AirComp MSE due to reduced signal strength at the server. Moreover, the shared power budget *P* introduces a trade-off between sensing (via $\textbf{F}_m$) and communication (via $\textbf{W}_m$), further complicating the optimization. These interactions suggest that overly strict constraints (e.g., very low $\Upsilon$, $\varepsilon _C$, or $\varepsilon _S$) might shrink the feasible region, potentially rendering the problem infeasible. To address this, our proposed smoothed exact penalty algorithm with CCCP iteratively adjusts the variables to balance these trade-offs, ensuring a feasible local optimum within practical parameter ranges.

Due to the coupling relationships between $\varvec{\Theta }$, $\textbf{A}$, ${{\textbf{W}}_{m}}$ and ${{\textbf{F}}_{m}}$, this problem lacks convexity. To ad-dress this issue, we propose an optimal design for the transmission beamforming.

Given the objective of minimizing AirComp MSE, it can be observed that both $\sum \limits _{m=1}^{M}{\operatorname {tr}}\left( \left( {{\textbf{A}}^{H}}{{\textbf{H}}_{m}}{{\textbf{W}}_{m}}-\textbf{I} \right) {{\left( {{\textbf{A}}^{H}}{{\textbf{H}}_{m}}{{\textbf{W}}_{m}}-\textbf{I} \right) }^{H}} \right)$ and $\sigma _{c}^{2}\operatorname {tr}\left( \textbf{A}{{\textbf{A}}^{H}} \right)$ are positive. Therefore, for any given data beamforming, the following inequality always holds:

42 $$\begin{aligned} \sum \limits _{m = 1}^M {{\mathop {{\textrm{tr}}}\nolimits } } \left( {\left( {{{\textbf{A}}^H}{{\textbf{H}}_m}{{\textbf{W}}_m} - {\textbf{I}}} \right) {{\left( {{{\textbf{A}}^H}{{\textbf{H}}_m}{{\textbf{W}}_m} - {\textbf{I}}} \right) }^H}} \right) + \sigma _c^2{\mathop {{\textrm{tr}}}\nolimits } \left( {{\textbf{A}}{{\textbf{A}}^H}} \right) \ge \sigma _c^2{\mathop {{\textrm{tr}}}\nolimits } \left( {{\textbf{A}}{{\textbf{A}}^H}} \right) \end{aligned}$$

Thus, it is straightforward to demonstrate that for a given data aggregation beam-former $\textbf{A}$, the zero-forcing transmission beamformer at the sensor minimizes the computation error, given by ${{\textbf{W}}_m} = {\left( {{\textbf{H}}_m^H{\textbf{A}}{{\textbf{A}}^H}{{\textbf{H}}_m}} \right) ^{ - 1}}{\textbf{H}}_m^H{\textbf{A}},\forall m$. The corresponding problem can therefore be formulated as:

43 $$\begin{aligned}&{\mathbf{(P2)}}\mathop {\min }\limits _{{\varvec{\Theta }},{\textbf{A}},\left\{ {{{\textbf{W}}_m}} \right\} ,\left\{ {{{\textbf{F}}_m}} \right\} } \sum \limits _{m = 1}^M {{\mathop {\textrm{tr}}\nolimits } } \left( {\left( {{{\textbf{A}}^H}{{\textbf{H}}_m}{{\textbf{W}}_m} - {\textbf{I}}} \right) {{\left( {{{\textbf{A}}^H}{{\textbf{H}}_m}{{\textbf{W}}_m} - {\textbf{I}}} \right) }^H}} \right) \\&+ \sum \limits _{m = 1}^M {{\mathop {\textrm{tr}}\nolimits } } \left( {{{\textbf{A}}^H}{{\textbf{R}}_m}{{\textbf{F}}_m}{\textbf{F}}_m^H{\textbf{R}}_m^H{\textbf{A}}} \right) + \sigma _c^2{\mathop {\textrm{tr}}\nolimits } \left( {{\textbf{A}}{{\textbf{A}}^H}} \right) \end{aligned}$$

43a $$\begin{aligned}&s.t.\quad {\mathop {\textrm{tr}}\nolimits } \left( {{{\left( {{{\textbf{F}}_m}{\textbf{F}}_m^H} \right) }^{ - 1}}} \right) \le \frac{{T{\eta _m}}}{{{N_{rr}}\sigma _r^2}},\forall m, \end{aligned}$$

43b $$\begin{aligned}&{\mathop {\textrm{tr}}\nolimits } \left( {{{\left( {{\textbf{H}}_m^H{\textbf{A}}{{\textbf{A}}^H}{{\textbf{H}}_m}} \right) }^{ - 1}}} \right) + {\mathop {\textrm{tr}}\nolimits } \left( {{{\textbf{F}}_m}{{\textbf{F}}_m}^H} \right) \le P,\forall m, \end{aligned}$$

43c $$\begin{aligned}&{\mathop {\textrm{CRB}}\nolimits } (\tilde{\varphi }) \le \Upsilon , \end{aligned}$$

43d $$\begin{aligned}&\mathscr {D}\left( \mathbb {P}_{0} \Vert \mathbb {P}_{1}\right) _{C} \le 2 \epsilon _{C}^{2} \end{aligned}$$

43e $$\begin{aligned}&\tilde{p}\left( \left\{ \textbf{F}_{m}\right\} , \textbf{S}\right) \le 2 \epsilon _{S} ^{2} \end{aligned}$$

Due to the coupling of variables $\varvec{\Theta }$, $\textbf{A}$, and ${{\textbf{F}}_{m}}$ in the objective function, problem (P2)(P2)(P2) is non-convex. Following the standard methods in MIMO beamforming literature^41–43^, the radar sensing beamformer ${{\textbf{F}}_{m}}$ is constrained to be an orthogonal matrix. Mathematically, this can be expressed as ${{\textbf{F}}_{m}}=\sqrt{{\textrm{B}_{m}}}{{\textbf{D}}_{m}}$, where ${{\textbf{D}}_{m}}$ is a unitary matrix, hence ${{\textbf{D}}_{m}}\textbf{D}_{m}^{H}=\textbf{I}$, and $\sqrt{{\textrm{B}_{m}}}$ is a positive scaling factor. The corresponding problem can be represented as:

44 $$\begin{aligned}&{\mathbf{(P3)}}\mathop {\min }\limits _{{\varvec{\Theta }},{\textbf{A}},\{ \mathrm{B_m}\} } \sum \limits _{m = 1}^M {\mathrm{B_m}} {\mathop {\textrm{tr}}\nolimits } \left( {{\textbf{R}}_m^H{\textbf{A}}{{\textbf{A}}^H}{{\textbf{R}}_m}} \right) + \sigma _c^2{\mathop {\textrm{tr}}\nolimits } \left( {{\textbf{A}}{{\textbf{A}}^H}} \right) \end{aligned}$$

44a $$\begin{aligned}&{\textrm{s}}{\mathrm{.t}}{\mathrm{. }}\quad \frac{{{N_{tx}}}}{{{\textrm{B}_m}}} \le \frac{{T{\eta _m}}}{{{N_{rx}}\sigma _r^2}},\forall m{\textrm{,}} \end{aligned}$$

44b $$\begin{aligned}&{\mathop {\textrm{tr}}\nolimits } \left( {{{\left( {{\textbf{H}}_m^H{\textbf{A}}{{\textbf{A}}^H}{{\textbf{H}}_m}} \right) }^{ - 1}}} \right) + {\textrm{B}_m} \le P,\forall m, \end{aligned}$$

44c $$\begin{aligned}&{\mathop {\textrm{CRB}}\nolimits } (\tilde{\varphi }) \le \Upsilon , \end{aligned}$$

44d $$\begin{aligned}&\mathscr {D}\left( \mathbb {P}_{0} \Vert \mathbb {P}_{1}\right) _{C} \le 2 \epsilon _{C}^{2} \end{aligned}$$

44e $$\begin{aligned}&\tilde{p}\left( \left\{ \textbf{F}_{m}\right\} , \textbf{S}\right) \le 2 \epsilon _{S} ^{2} \end{aligned}$$

It can be observed that an increase in ${\textrm{B}_{m}}$ leads to an increase in MSE. Therefore, when using minimal $\mathrm B_{m}^{*}$ for all ${\textrm{B}_{m}}$, the MSE is minimized, given by $\alpha _m^* = \frac{{{N_{rt}}{N_{rr}}\sigma _r^2}}{{T{\eta _m}}},\forall m$. By introducing $\mathbf {\hat{A}}=\textbf{A}{{\textbf{A}}^{H}}$ and applying Semidefinite Relaxation (SDR), the problem can be represented as:

45 $$\begin{aligned}&{\mathbf{(P4)}}\mathop {\min }\limits _{{\varvec{\Theta }},{\mathbf{{\hat{A}}}}} \sum \limits _{m = 1}^M {\frac{{{N_{tx}}{N_{rx}}\sigma _r^2}}{{T{\eta _m}}}} {\mathop {\textrm{tr}}\nolimits } \left( {{\textbf{R}}_m^H\widehat{\textbf{A}}{{\textbf{R}}_m}} \right) + \sigma _c^2{\mathop {\textrm{tr}}\nolimits } (\widehat{\textbf{A}}) \end{aligned}$$

45a $$\begin{aligned}&{\textrm{s}}{\mathrm{.t}}{\mathrm{. }}\quad {\mathop {\textrm{tr}}\nolimits } \left( {{{\left( {{\textbf{H}}_m^H\widehat{\textbf{A}}{{\textbf{H}}_m}} \right) }^{ - 1}}} \right) + \frac{{{N_{rt}}{N_{tt}}\sigma _r^2}}{{T{\eta _m}}} \le P,\forall m, \end{aligned}$$

45b $$\begin{aligned} \widehat{\textbf{A}} \ge 0, \end{aligned}$$

45c $$\begin{aligned}&{\mathop {\textrm{CRB}}\nolimits } (\tilde{\varphi }) \le \Upsilon , \end{aligned}$$

45d $$\begin{aligned}&\mathscr {D}\left( \mathbb {P}_{0} \Vert \mathbb {P}_{1}\right) _{C} \le 2 \epsilon _{C}^{2}, \end{aligned}$$

45e $$\begin{aligned}&\tilde{p}\left( \left\{ \textbf{F}_{m}\right\} , \textbf{S}\right) \le 2 \epsilon _{S} ^{2}. \end{aligned}$$

Next, we address the covert communication constraint ${\mathscr {D}}{\left( {{\mathbb {P}_0}{\mathbb {P}_1}} \right) _C} = M\left[ {\ln \left( {\frac{{{{(p_m^C)}^2}{{\left| {{{\textbf{H}}_{m,{W_C}}}} \right| }^2} + \sigma _w^2}}{{\sigma _w^2}}} \right) } \right. \left. { + \frac{{\sigma _w^2}}{{{{(p_m^C)}^2}{{\left| {{{\textbf{H}}_{m,{W_C}}}} \right| }^2} + \sigma _w^2}} - 1} \right]$, and introduce a new variable, denoted as $x\triangleq \frac{{{\sigma }_{1}}}{{{\sigma }_{0}}}=\frac{{{(p_{m}^{C})}^{2}}{{\left| {{\textbf{H}}_{m,{{W}_{C}}}} \right| }^{2}}+\sigma _{w}^{2}}{\sigma _{w}^{2}}$. Therefore, the constraint ${\mathscr {D}}{{\left( {{\mathbb {P}}_{0}}\Vert {{\mathbb {P}}_{1}} \right) }_{C}}\le 2{{{\epsilon }}_{C}}^{2}$ can be reformulated as:

46 $$\begin{aligned} f(x) \triangleq \ln x+\frac{1}{x} \le 1+2 \epsilon _{C} ^{2} \end{aligned}$$

By introducing $x_1$ and $x_2$ as the two roots of $f(x) = 1 + 2\varepsilon _C^2$, where $f(x) = \ln x + \frac{1}{x}$ is an increasing function over $\left[ x_1, x_2\right]$, we transform $\mathscr {D}\left( \mathbb {P}_0 | \mathbb {P}1\right) C \le 2\varepsilon _C^2$ into $x_1 \le x \le x_2$. Here, $x_1 =$ $\exp \left( \mathscr {W}_{-1}\left( -\exp \left( -\left( 1 + 2\varepsilon _C^2\right) \right) \right) + 1 + 2\varepsilon _C^2\right)$ and $x_2 =$ $\exp \left( \mathscr {W}_0\left( -\exp \left( -\left( 1 + 2\varepsilon _C^2\right) \right) \right) + 1 + 2\varepsilon _C^2\right)$, with $\mathscr {W}_{-1}$ and $\mathscr {W}_0$ denoting the −1 and 0 branches of the Lambert $\mathscr {W}$ function, respectively. The argument of $\mathscr {W}{-1}$, $z = -\exp \left( -\left( 1 + 2\varepsilon _C^2\right) \right)$, must satisfy $-\frac{1}{e} \le z < 0$ for $\mathscr {W}{-1}$ to be defined. Since $\varepsilon _C^2 \ge 0$ and typically small in covert systems (e.g., $0 < \varepsilon _C \le \sqrt{\frac{\ln 2}{2}}$), we have $-\exp (-1) \le z < 0$, ensuring $z \ge -\frac{1}{e}$ when $\varepsilon _C^2 \le \frac{\ln 2}{2}$. For consistency, we assume $\varepsilon _C^2 \le \frac{\ln 2}{2}$ in this work, which aligns with practical covertness requirements and keeps *z* within the valid domain. Given that $x=1+\frac{{{(p_{m}^{C})}^{2}}{{\left| {{\textbf{H}}_{m,{{W}_{C}}}} \right| }^{2}}}{\sigma _{w}^{2}}>1$, we obtain $0< \frac{{{{(p_m^C)}^2}{{\left| {{{\textbf{H}}_{m,{W_C}}}} \right| }^2}}}{{\sigma _w^2}} < {x_2} - 1$.Thus, problem (P4) can be rewritten as:

47 $$\begin{aligned}&{\mathbf{(P5)}}\mathop {\min }\limits _{{\varvec{\Theta }},{\mathbf{{\hat{A}}}}} \sum \limits _{m = 1}^M {\frac{{{N_{tx}}{N_{rx}}\sigma _r^2}}{{T{\eta _m}}}} {\mathop {\textrm{tr}}\nolimits } \left( {{\textbf{R}}_m^H\widehat{\textbf{A}}{{\textbf{R}}_m}} \right) + \sigma _c^2{\mathop {\textrm{tr}}\nolimits } (\widehat{\textbf{A}}) \end{aligned}$$

47a $$\begin{aligned}&{\textrm{s}}{\mathrm{.t}}{\mathrm{. }}\quad {\mathop {\textrm{tr}}\nolimits } \left( {{{\left( {{\textbf{H}}_m^H\widehat{\textbf{A}}{{\textbf{H}}_m}} \right) }^{ - 1}}} \right) + \frac{{{N_{rt}}{N_{tt}}\sigma _r^2}}{{T{\eta _m}}} \le P,\forall m, \end{aligned}$$

47b $$\begin{aligned}&\widehat{\textbf{A}} \ge 0, \end{aligned}$$

47c $$\begin{aligned}&{\mathop {\textrm{CRB}}\nolimits } (\tilde{\varphi }) \le \Upsilon , \end{aligned}$$

47d $$\begin{aligned}&0< \frac{\left( p_m^C\right) ^2\left| \textbf{H}{m, W_C}\right| ^2}{\sigma _w^2}< x_2 - 1, \text {with} 0 < \varepsilon _C^2 \le \frac{\ln 2}{2}, \end{aligned}$$

47e $$\begin{aligned} \tilde{p}\left( \left\{ \textbf{F}_{m}\right\} , \textbf{S}\right) \le 2 \epsilon _{S} ^{2}. \end{aligned}$$

Given $\varvec{\Theta }$, the optimization in problem (47) can be reduced to an unconstrained optimization problem, denoted as $\underset{\widehat{\textbf{A}}}{\mathop {\min }}\,\text {MS}{{\text {E}}_{\widehat{\textbf{A}}}}$. Based on the principles of optimal Minimum Mean Squared Error (MMSE) receiver design, the optimal solution for $\widehat{\textbf{A}}$ is expressed as ${\textbf{U}}_a^o = {\left( {{\textbf{A}} + \sigma _c^2{\mathop {\textrm{tr}}\nolimits } (\widehat{\textbf{A}})} \right) ^{ - 1}}{\textbf{C}}$. where, ${\textbf{A}} = \sum \limits _{m = 1}^M {{\textbf{R}}_m^H{{\textbf{R}}_m}}$, ${\textbf{C}} = \sum \limits _{m = 1}^M {{{\textbf{R}}_m}}$. Additionally, we set $d = \sum \limits _{m = 1}^M {{{(p_m^C)}^2}{{\left| {{{\textbf{H}}_{m,{W_C}}}} \right| }^2}}$. The constraint (52 d) can be rewritten $d \le \left( {{x_2} - 1} \right) \left( {\sigma _w^2} \right)$, Substituting Equations ${\textbf{U}}_a^o$ and *d* into Equation $0<\frac{{{(p_{m}^{C})}^{2}}{{\left| {{\textbf{H}}_{m,{{W}_{C}}}} \right| }^{2}}}{\sigma _{w}^{2}}<{{x}_{2}}-1$, problem (P5) can be formulated as:

48 $$\begin{aligned}&{\mathbf{(P6)}}\mathop {\min }\limits _{{\varvec{\Theta }},{\mathbf{{\hat{A}}}}} \sum \limits _{m = 1}^M {\frac{{{N_{tx}}{N_{rx}}\sigma _r^2}}{{T{\eta _m}}}} {\mathop {\textrm{tr}}\nolimits } \left( {{\textbf{R}}_m^H\widehat{\textbf{A}}{{\textbf{R}}_m}} \right) + \sigma _c^2{\mathop {\textrm{tr}}\nolimits } (\widehat{\textbf{A}}) \end{aligned}$$

48a $$\begin{aligned}&{\textrm{s}}{\mathrm{.t}}{\mathrm{. }}\quad {\mathop {\textrm{tr}}\nolimits } \left( {{{\left( {{\textbf{H}}_m^H\widehat{\textbf{A}}{{\textbf{H}}_m}} \right) }^{ - 1}}} \right) + \frac{{{N_{rt}}{N_{tt}}\sigma _r^2}}{{T{\eta _m}}} \le P,\forall m, \end{aligned}$$

48b $$\begin{aligned}&\widehat{\textbf{A}} \ge 0, \end{aligned}$$

48c $$\begin{aligned}&{\mathop {\textrm{CRB}}\nolimits } (\tilde{\varphi }) \le \Upsilon , \end{aligned}$$

48d $$\begin{aligned}&{\textbf{A}} = \sum \limits _{m = 1}^M {{\textbf{R}}_m^H{{\textbf{R}}_m}} , \end{aligned}$$

48e $$\begin{aligned}&{\textbf{C}} = \sum \limits _{m = 1}^M {{{\textbf{R}}_m}}, \end{aligned}$$

48f $$\begin{aligned}&d = \sum \limits _{m = 1}^M {{{(p_m^C)}^2}{{\left| {{{\textbf{H}}_{m,{W_C}}}} \right| }^2}}, \end{aligned}$$

48g $$\begin{aligned}&\tilde{p}\left( \left\{ \textbf{F}_{m}\right\} , \textbf{S}\right) \le 2 \epsilon _{S} ^{2}. \end{aligned}$$

where $\Gamma =\frac{{{N}_{tx}}{{N}_{rx}}\sigma _{r}^{2}}{T{{\eta }_{m}}}$, *K* represent the number of sensors, and ${{N}_{s}}$ represents the number of antennas equipped on the sensors. Since the objective function (48) and the equality constraints (48 d), (48e), (48f) are non-convex, problem (P5) remains non-convex. Therefore, finding a global optimal solution to optimization problem (58) is challenging. We can transform (48 d), (48e), and (48f) into appropriate convex forms, allowing us to obtain a local optimal solution using the constrained concave-convex procedure (CCCP).

Assuming $\textbf{Y}\succ \textbf{0}$, $\varvec{\Omega }={{\textbf{X}}^{H}}{{\textbf{Y}}^{-1}}\textbf{X}$ is equivalent to:

49 $$\begin{aligned} \left[ \begin{array}{cc} \varvec{\Omega } & \textbf{X}^{H} \\ \textbf{X} & \textbf{Y} \end{array}\right] \succeq \textbf{0} \end{aligned}$$

and $${\mathop {\textrm{tr}}\nolimits } \left( {{\varvec{\Omega }} - {{\textbf{X}}^H}{{\textbf{Y}}^{ - 1}}{\textbf{X}}} \right) \le 0$$.

Let us define auxiliary variables $\textbf{T}$ and $\textbf{s}$ as:

50 $$\begin{aligned} {\textbf{T}} = \left[ {{{\textbf{R}}_1}, \cdots ,{{\textbf{R}}_M}} \right] \end{aligned}$$

51 $$\begin{aligned} {\textbf{s}} = \left[ {{{(p_1^C)}^2}{{\left| {{{\textbf{H}}_{1,{W_C}}}} \right| }^2}, \cdots ,{{(p_K^C)}^2}{{\left| {{{\textbf{H}}_{K,{W_C}}}} \right| }^2}} \right] \end{aligned}$$

The equality constraints (48 d) and (48f) can be equivalently represented as:

52 $$\begin{aligned} & \left[ \begin{array}{cc} \textbf{A} & \textbf{T} \\ \textbf{T}^{H} & \textbf{I} \end{array}\right] \succeq \textbf{0} \end{aligned}$$

53 $$\begin{aligned} & {\mathop {\textrm{tr}}\nolimits } \left( {{{\textbf{A}}_a} - {\textbf{T}}{{\textbf{T}}^H}} \right) \le 0 \end{aligned}$$

54 $$\begin{aligned} & \left[ \begin{array}{cc} d & \textbf{s} \\ \textbf{s}^{H} & \textbf{I} \end{array}\right] \succeq \textbf{0} \end{aligned}$$

55 $$\begin{aligned} & {\mathop {\textrm{tr}}\nolimits } \left( {d - {\textbf{S}}{{\textbf{S}}^H}} \right) \le 0 \end{aligned}$$

Thus, problem (P6) can be reformulated as:

56 $$\begin{aligned} \mathop {\min }\limits _\Xi K{N_s} - {\mathop {\textrm{tr}}\nolimits } \left( {{{\textbf{C}}^H}{{\left( {{\textbf{A}} + {\textbf{B}} + \sigma _a^2{{\textbf{I}}_{{N_r}}}} \right) }^{ - 1}}{\textbf{C}}} \right) \nonumber \\ {\textrm{s}}{\mathrm{.t}}{\mathrm{. (48a),(48b),(48c), (48e),(48f),(48g),(50) - (55)}} \end{aligned}$$

where $\Xi =\left\{ {{\textbf{W}}_{k}},\textbf{V},\textbf{T},\textbf{s},\textbf{A},\textbf{B},\textbf{C},d,e \right\}$ in problem (56), constraints (52) and 67 are linear matrix inequalities. Constraints (58) and (48f) are linear. However, the objective function (56), constraints (53), and (55) remain non-convex and exhibit a Difference of Convex (DC) structure. To solve problem (41), we first transform problem (41) into a DC programming problem and then use a penalty-based algorithm to obtain a local optimal solution.

Thus, define $\Upsilon \triangleq \{\textbf{A},\textbf{C}\}$, $\Gamma \triangleq \{\textbf{A},d\}$, $\Psi \triangleq \{\textbf{T},\textbf{s}\}$ along with the function:

57 $$\begin{aligned} f(\Upsilon )= & {\mathop {\textrm{tr}}\nolimits } \left( {{{\textbf{C}}^H}{{\left( {{\textbf{A}} + \sigma _a^2{{\textbf{I}}_{{N_r}}}} \right) }^{ - 1}}{\textbf{C}}} \right) \end{aligned}$$

58 $$\begin{aligned} f(\Gamma )= & {\mathop {\textrm{tr}}\nolimits } ({\textbf{A}} + d) \end{aligned}$$

59 $$\begin{aligned} f(\Psi )= & {\mathop {\textrm{tr}}\nolimits } \left( {{\textbf{T}}{{\textbf{T}}^H} + {\textbf{s}}{{\textbf{s}}^H}} \right) \end{aligned}$$

Using a penalty-based exact algorithm, we can reformulate problem (69) as:

60 $$\begin{aligned} \mathop {\min }\limits _{\Xi \in \Theta } K{N_s} - f(\Upsilon ) + p(f(\Gamma ) - f(\Psi )) \end{aligned}$$

$\text{where}:\,\buildrel \Delta \over = \{ \Xi \mid {\mathrm{(48a),(48b),(48c), (48e),(48f),(48g),(50) - (55),(54)}}\}$, *p* represents the penalty factor. We can then solve problem (73) using a CCCP-based algorithm. The first-order Taylor series expansions of $f(\Upsilon )$ and $f(\Psi )$ around points $\bar{\Upsilon }$ and $\bar{\Psi }$ are given by:

61 $$\begin{aligned} & \begin{array}{l} f(\Upsilon ;\bar{\Upsilon }) = - {\mathop {\textrm{tr}}\nolimits } \left( {{{\overline{\textbf{C}} }^H}{{\left( {\overline{\textbf{A}} + \sigma _a^2{{\textbf{I}}_{{N_r}}}} \right) }^{ - 1}}\overline{\textbf{C}} } \right) + 2\mathbb {R}\left\{ {{\mathop {\textrm{tr}}\nolimits } \left( {{{\overline{\textbf{C}} }^H}{{\left( {\overline{\textbf{A}} + \sigma _a^2{{\textbf{I}}_{{N_r}}}} \right) }^{ - 1}}{\textbf{C}}} \right) } \right\} \\ -\mathbb {R} \left\{ {tr \left( {{{\overline{\textbf{C}} }^H}{{\left( {\overline{\textbf{A}} + \sigma _a^2{{\textbf{I}}_{{N_r}}}} \right) }^{ - 1}}({\textbf{A}} - \overline{\textbf{A}} )} \right. } \right. \left. {\left. { \cdot {{\left( {\overline{\textbf{A}} + \sigma _a^2{{\textbf{I}}_{{N_r}}}} \right) }^{ - 1}}\overline{\textbf{C}} } \right) } \right\} \end{array} \end{aligned}$$

62 $$\begin{aligned} & \begin{array}{l} f(\Psi ;\bar{\Psi }) = - {\mathop {\textrm{tr}}\nolimits } \left( {\overline{\textbf{T}} {{\overline{\textbf{T}} }^H} + \overline{\textbf{s}} {{\overline{\textbf{s}} }^H}} \right) + 2\mathbb {R}\left\{ {{{\textrm{tr}}}\left( {{\textbf{T}}{{\overline{\textbf{T}} }^H} + {\textbf{s}}{{\overline{\textbf{s}} }^H}} \right) } \right\} \end{array} \end{aligned}$$

Assuming $\left( {{\Upsilon }^{(m)}},{{\Psi }^{(m)}} \right)$ is optimal in the m-th iteration, problem (60) can be formulated as:

63 $$\begin{aligned} \mathop {\min }\limits _{\Xi \in \Theta } K{N_s} - f\left( {\Upsilon ;{\Upsilon ^{(m)}}} \right) + p\left( {f(\Gamma ) - f\left( {\Psi ;{\Psi ^{(m)}}} \right) } \right) \end{aligned}$$

where the optimal $\textbf{A}$,$\textbf{C}$,$\textbf{d}$,$\textbf{e}$,$\textbf{s}$,${{\textbf{W}}_{k}}$,$\textbf{T}$,$\textbf{V}$ in the m-th iteration are denoted as ${{\textbf{A}}^{\left( m \right) }}$,${{\textbf{B}}^{\left( m \right) }}$,${{\textbf{C}}^{\left( m \right) }}$,${{\textbf{d}}^{\left( m \right) }}$,${{\textbf{e}}^{\left( m \right) }}$,${{\textbf{s}}^{\left( m \right) }}$,$\textbf{W}_{k}^{\left( m \right) }$,${{\textbf{T}}^{\left( m \right) }}$,${{\textbf{V}}^{\left( m \right) }}$

To address the optimization problem, we propose a CCCP-based optimization algorithm, with its detailed steps outlined in Table 1. The algorithm iteratively updates the variables ${{\textbf{A}}^{\left( m \right) }}$,${{\textbf{B}}^{\left( m \right) }}$,${{\textbf{C}}^{\left( m \right) }}$,${{\textbf{d}}^{\left( m \right) }}$,${{\textbf{e}}^{\left( m \right) }}$,${{\textbf{s}}^{\left( m \right) }}$,$\textbf{W}_{k}^{\left( m \right) }$,${{\textbf{T}}^{\left( m \right) }}$,${{\textbf{V}}^{\left( m \right) }}$ by solving Equation (63), as summarized in Table 1. The detailed steps of the CCCP-based optimization algorithm are shown in Algorithm 1, e.g. Table 1.

**Table 1:** CCCP-Based Optimization Algorithm.

Algorithm 1: CCCP-Based Optimization Algorithm	
1: Initialization:$m=0$,${{\textbf{A}}^{\left( 0 \right) }}$,${{\textbf{B}}^{\left( 0 \right) }}$,${{\textbf{C}}^{\left( 0 \right) }}$,${{\textbf{s}}^{\left( 0 \right) }}$,${{\textbf{T}}^{\left( 0 \right) }}$,${{\textbf{V}}^{\left( 0 \right) }}$;	
2: repeat	
$$m=m+1$$;	
Update $\textbf{W}_{k}^{\left( m \right) }$,${{\textbf{T}}^{\left( m \right) }}$,${{\textbf{V}}^{\left( m \right) }}$,${{\textbf{A}}^{\left( m \right) }}$,${{\textbf{B}}^{\left( m \right) }}$,${{\textbf{C}}^{\left( m \right) }}$,${{\textbf{s}}^{\left( m \right) }}$,${{\textbf{d}}^{\left( m \right) }}$,${{\textbf{e}}^{\left( m \right) }}$ by solving Equation (63).	
3: Until Algorithm convergence.	

## Simulation results and analysis

In this section, we evaluate the performance of the proposed covert ISCCO framework through simulations. The simulations are based on the radar sensing and AirComp channel models described in equations (4), (6), (7), and (8). The performance metric is the normalized AirComp MSE, defined as MSE/M, where the AirComp MSE is given in equation (8). Unless otherwise specified, the simulation parameters are set as follows:

- The number of time slots: $T=1000$.
- The number of computation functions: $K=10$.
- The number of sensors: $M=10$, each equipped with ${{N}_{s}}=4$ antennas.
- One AP equipped with $M=64$ antennas.
- Among these, ${{N}_{c}}=32$ antennas are used for data transmission, and ${{N}_{r}}=32$ antennas are used for radar sensing. Specifically, ${{N}_{rt}}=16$ antennas are for radar signal transmission, and ${{N}_{rr}}=16$ antennas are for radar signal reception.
- All channels are assumed to be i.i.d. fading channels, modeled as i.i.d. complex Gaussian random variables with non-zero mean $\mu =1$ and variance ${{\sigma }^{2}}=1$.
- The maximum transmission power is set to ${{P}_{0}}=10mw$.
- The effective power conversion efficiency follows a uniform distribution ${{\eta }_{n}}\in \left( 0,1 \right)$.
- According to the LTE settings^44^, the noise power for the radar signal channel $\sigma _{r}^{2}$ and the data transmission channel $\sigma _{c}^{2}$ is −79.5 dBm.
- Each point in the figures is obtained by averaging multiple simulation realizations, with independent channels in each realization.

The covert communication threshold $\varepsilon _C$ is set within $0 < \varepsilon _C \le \sqrt{\frac{\ln 2}{2}} \approx 0.589$ to ensure the argument of $\mathscr {W}_{-1}$ remains within its valid domain, consistent with the theoretical derivation.

The simulations assume perfect CSI at all nodes and eavesdroppers to validate the proposed optimization framework under ideal conditions. Due to the complexity of the system model, this assumption simplifies the analysis but may deviate from real-world scenarios. Future studies will explore the impact of imperfect CSI on AirComp MSE, sensing accuracy, and covertness. Moreover, the perfect CSI assumption for eavesdroppers simulates the most stringent eavesdropping environment, ensuring that the optimization results demonstrate robustness under the worst-case conditions.

The Rayleigh fading model is adopted in these simulations to represent a challenging NLOS environment, where the absence of a LOS component leads to significant signal variations, making it suitable for evaluating the robustness of the STAR-RIS-assisted ISCCO system. The zero-mean, unit-variance complex Gaussian distribution ($\mathscr{C}\mathscr{N}(0, 1)$) for channel coefficients ensures that performance metrics, such as AirComp MSE and covertness, are tested under diverse multi-path conditions. However, this model may not fully capture scenarios with a strong LOS path (e.g., open areas), where a Rician fading model with a non-zero mean and K-factor would be more appropriate. Future work could explore Rician fading to assess the system’s performance under varying LOS strengths, potentially enhancing its applicability to diverse deployment settings.

**Fig. 2:**
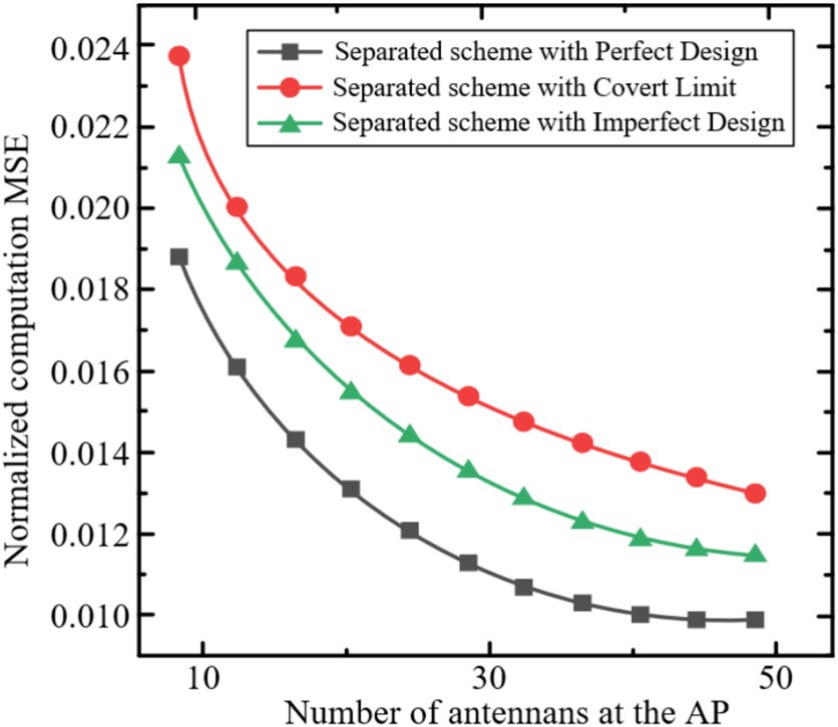
The Relationship Between Normalized AirComp MSE and the Number of AP Antennas.

Figure 2 evaluates the relationship between the normalized AirComp MSE and the number of AP antennas. As observed, the normalized AirComp MSE decreases as the number of AP antennas increases. This is because the increase in the number of AP antennas enhances the dimensionality of the data aggregation beamformer, leveraging diversity gain to achieve a lower AirComp MSE. It is noteworthy that after incorporating the covert communication or covert sensing constraints, the AirComp MSE experiences a slight reduction. This is because some AirComp MSE performance is sacrificed to ensure covert performance under these constraints.

**Fig. 3:**
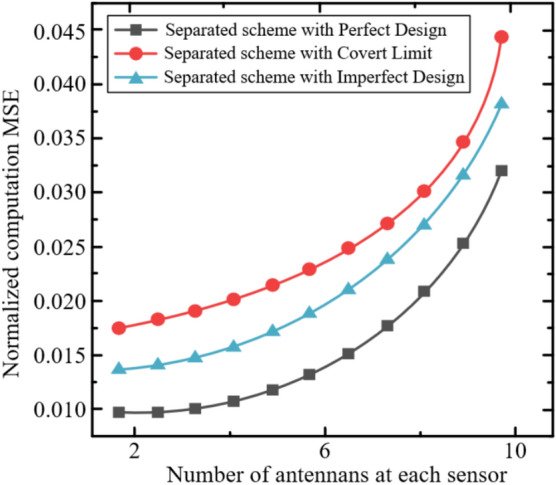
The Relationship Between Normalized AirComp MSE and the Number of Antennas per Sensor.

Figure 3 shows the relationship between the normalized AirComp MSE and the number of antennas per sensor under both shared and separate schemes. It can be observed that the normalized AirComp MSE increases monotonically with the number of antennas per sensor. This is because more antennas on the sensor increase the TRM size that needs to be estimated, thereby tightening the sensing constraints. Consequently, the beamformer needs to ensure radar sensing requirements at the expense of AirComp performance. Furthermore, as the number of antennas per sensor increases, the shared scheme outperforms the separate scheme. This phenomenon is attributed to the dual effect of deploying more antennas on each sensor. On one hand, more antennas on each sensor enlarge the dimension of the beamforming matrix, which supports the dual functionality of signals in the shared scheme. On the other hand, in the separate scheme, more antennas on each sensor for radar sensing exacerbate interference with AirComp. A similar trend is observed for the curves under the perfect design.

**Fig. 4:**
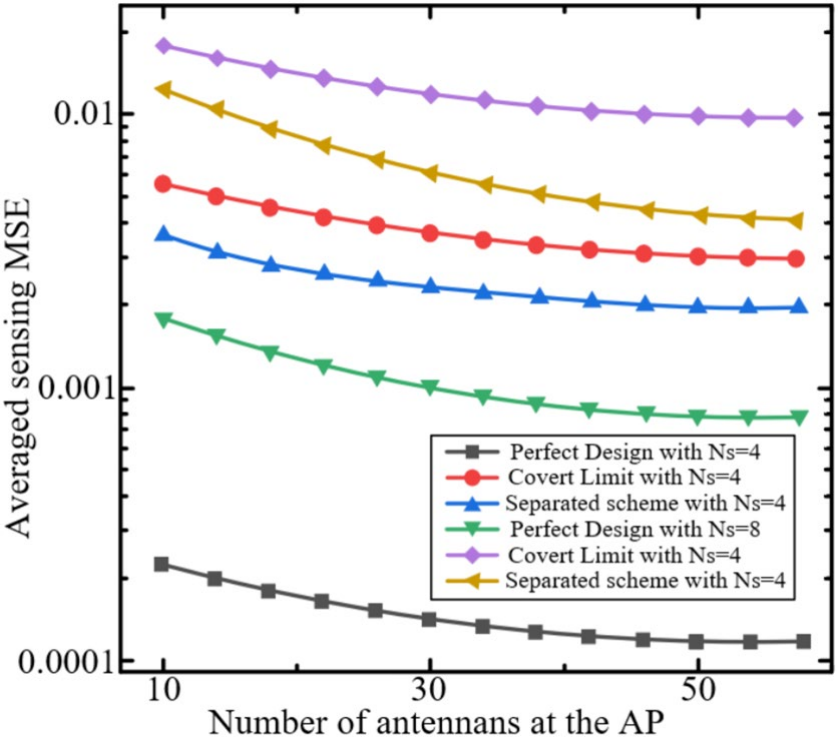
The Impact of the Number of Antennas on the Average Sensing MSE.

For radar sensing, the impact of the number of antennas at the sensors and AP on the average sensing MSE is shown in Figure 4. It can be observed that in the shared scheme, the average sensing MSE decreases as the number of antennas at the AP increases, indicating that increasing the size of the aggregation beamformer provides greater flexibility in achieving a lower sensing MSE. Additionally, in the shared scheme, deploying more antennas on each sensor leads to a higher average sensing MSE because the TRM size to be estimated increases. In contrast, in the separate scheme, the average sensing MSE at the AP and sensors does not change with the number of antennas, as the radar sensing constraints are more stringent to mitigate the interference of radar signals with AirComp. Therefore, the sensing MSE depends solely on the sensing quality requirements, independent of other parameters.

**Fig. 5:**
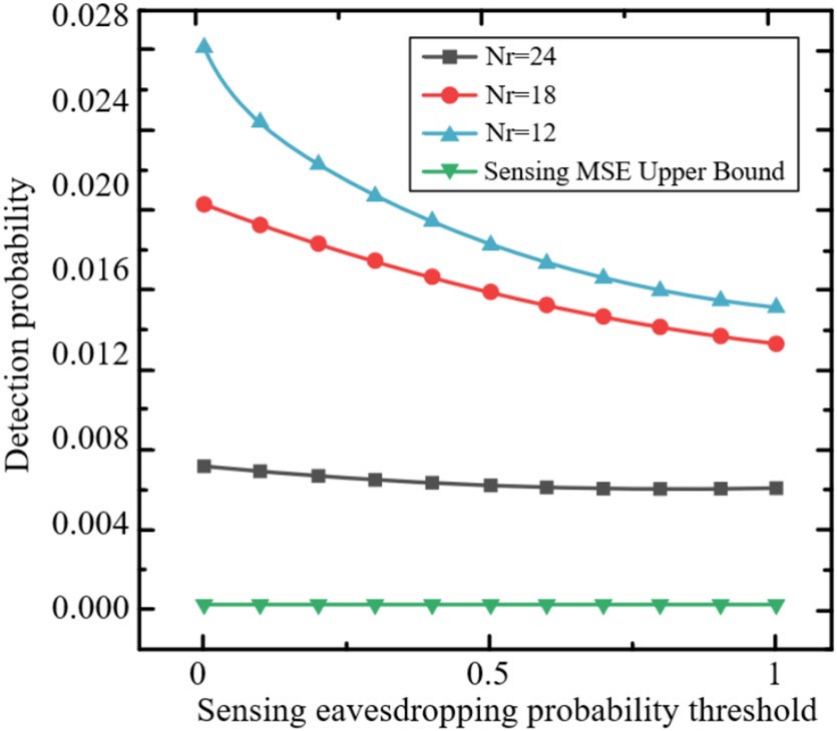
The Impact of Eavesdropping Probability Threshold on Sensing MSE.

Figure 5 presents the relationship between the eavesdropper’s detection probability and the sensing eavesdropping probability threshold $\varepsilon _S^2$ under different numbers of radar antennas ($N_r = 12, 18, 24$) when the power budget is $P_0 = 10 , \text {mW}$. It is evident that the looser the sensing eavesdropping probability constraint or the larger the $\varepsilon _S^2$ value, the higher the detection probability, indicating that covert performance is compromised as the constraint is relaxed. Specifically, the detection probability increases from approximately 0.00 to 0.024 as $\varepsilon _S^2$ rises from 0 to 1.5 for $N_r = 24$. It is also observed that as the number of radar antennas increases, the detection probability decreases for a given $\varepsilon _S^2$; for instance, at $\varepsilon _S^2 = 1$, the detection probability drops from 0.20 for $N_r = 12$ to 0.004 for $N_r = 24$, reflecting improved covert sensing due to enhanced beamforming precision with more antennas. The sensing MSE upper bound remains constant at 0.00, serving as a reference for the best-case covert performance.

**Fig. 6:**
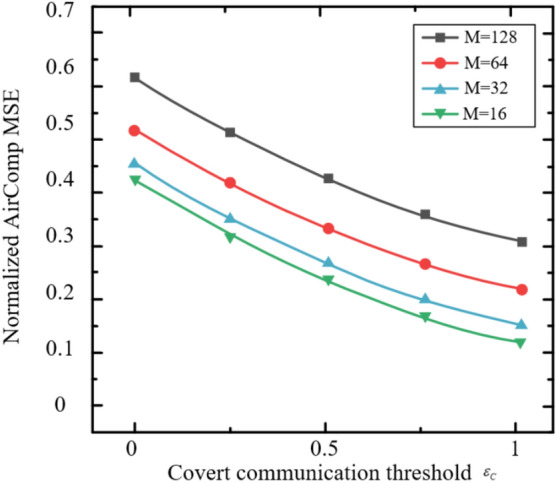
The Trade-Off Between Normalized AirComp MSE and Covert Communication Threshold $\varepsilon _C$.

To further explore the interactions between the CRB threshold and covert constraints, we evaluate the feasibility of the optimization framework under varying constraint tightness. Figure 6 illustrates the normalized AirComp MSE versus the covert communication threshold $\varepsilon _C$ for different CRB thresholds $\Upsilon$ (e.g., $\Upsilon = 0.01, 0.05, 0.1$), with $\varepsilon _S = 0.1$ and $P_0 = 10 , \text {mW}$. As $\varepsilon _C$ decreases (stricter covertness), the MSE increases due to reduced power allocation to $\textbf{W}_m$, and for a stringent $\Upsilon = 0.01$, the MSE rises more sharply, indicating a tighter feasible region. When $\varepsilon _C < 0.05$ and $\Upsilon = 0.01$, the problem becomes infeasible, as no solution satisfies all constraints simultaneously. Conversely, with a looser $\Upsilon = 0.1$, the framework remains feasible across a wider range of $\varepsilon _C$, demonstrating that relaxing one constraint can mitigate the impact of another. These results confirm that while constraint interactions can challenge feasibility under extreme conditions, the proposed algorithm effectively navigates these trade-offs within practical settings, as validated by the convergence observed in Figures 2–5.

Figure 6 plots the normalized AirComp MSE against the covert communication threshold $\varepsilon _C$ for different numbers of AP antennas ($M = 16$, $M = 32$, $M = 64$, and $M = 128$), with $P_0 = 10 , \text {mW}$ and $\varepsilon _S = 0.1$, illustrating the trade-off between covert communication and computational accuracy. As $\varepsilon _C$ increases from 0 to 1.5, the normalized AirComp MSE decreases across all *M* values, e.g., from 0.38 to 0.13 for $M = 128$, indicating that a looser covert constraint allows more power allocation to $\textbf{W}_m$, enhancing computational accuracy at the cost of covertness. Moreover, increasing *M* significantly reduces MSE; for instance, at ${{\varepsilon }_{C}}=1$, MSE drops from 0.27 for $M = 16$ to 0.13 for $M = 128$, a reduction of approximately 52%, due to improved signal aggregation with more AP antennas, underscoring the advantage of massive MIMO in optimizing this trade-off.

**Fig. 7:**
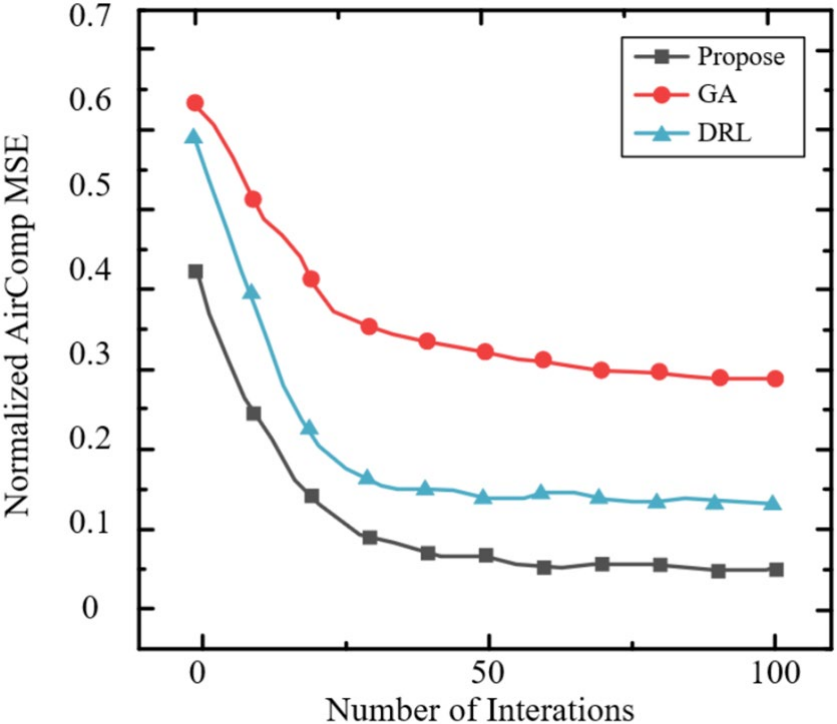
Performance Comparison with Cutting-Edge Algorithms Over Iterations.

Figure 7 compares the normalized AirComp MSE of our proposed smoothed exact penalty algorithm with CCCP against two cutting-edge approaches-a genetic algorithm (GA) and a deep reinforcement learning (DRL) method-over iterations, under the settings $M = 10$, $N_s = 4$, $N = 16$, $P_0 = 10 , \text {mW}$, $\varepsilon _C = 0.1$, and $\varepsilon _S = 0.1$. The proposed method converges rapidly, reducing MSE from 0.30 to 0.18 within 20 iterations, demonstrating efficient optimization of non-convex constraints like covertness. In contrast, the GA requires significantly more iterations, reaching an MSE of 0.25 after 100 iterations, due to its slower convergence in handling complex constraints. The DRL method achieves a slightly better MSE of 0.16 but requires 50 episodes (interpreted as iterations here) to stabilize, reflecting its high computational cost during training. This comparison underscores the proposed algorithm’s superior convergence speed and efficiency, making it more suitable for real-time ISAC applications, while DRL’s performance suggests potential for hybrid approaches in future work.

**Fig. 8:**
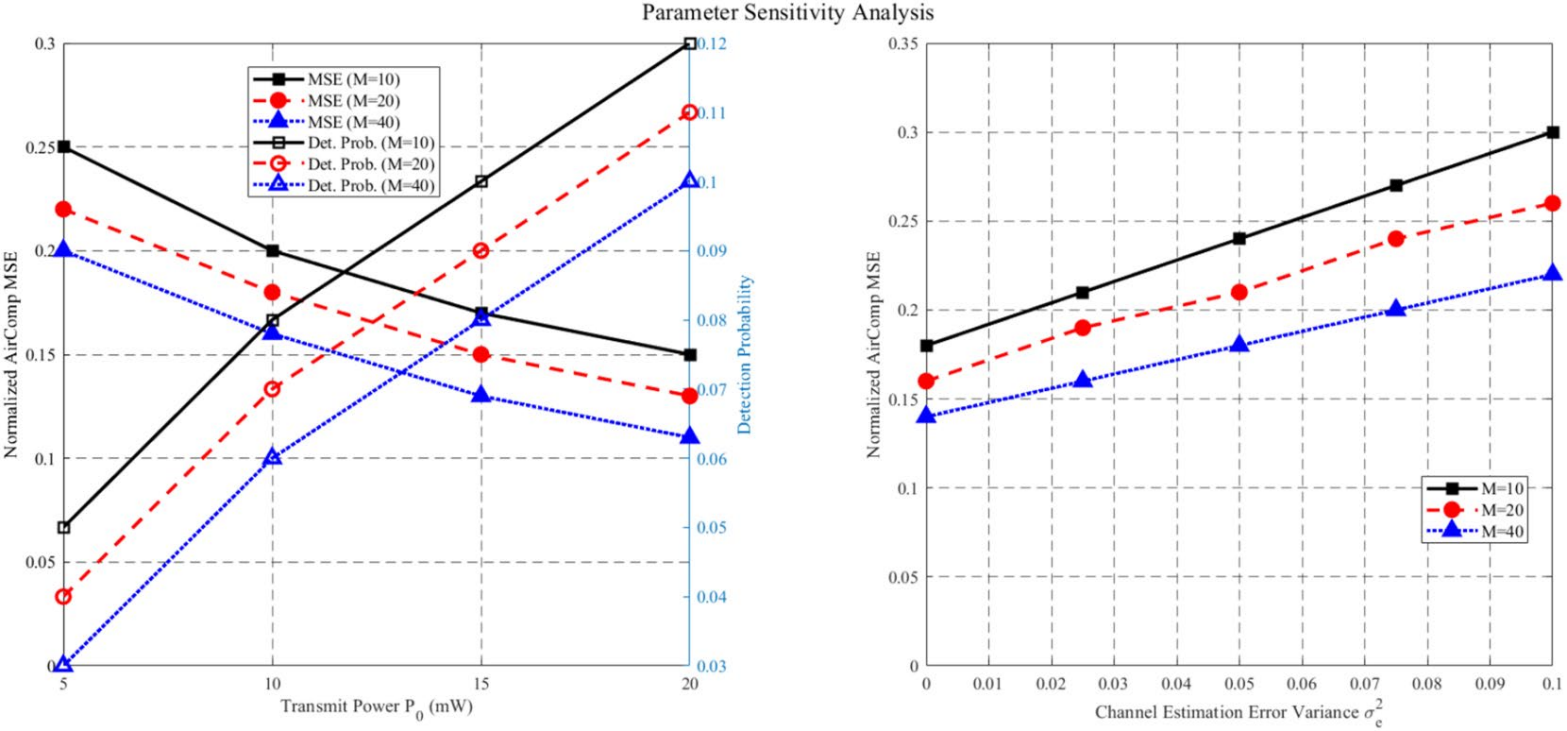
Parameter Sensitivity Analysis.

Figure 8 evaluates the impact of transmit power ($P_0$) and channel estimation error on system performance for different numbers of AP antennas ($M = 10$, $M = 20$, $M = 40$), with $N_s = 4$, $N = 16$, $\varepsilon _C = 0.1$, and $\varepsilon _S = 0.1$. Subfigure (a) plots the normalized AirComp MSE and detection probability versus $P_0$ (5 to 20 mW). As $P_0$ increases, MSE decreases for all *M* values, e.g., from 0.25 to 0.15 for $M = 10$, and from 0.20 to 0.11 for $M = 40$, due to improved SNR enhancing computation accuracy; however, detection probability rises, e.g., from 0.05 to 0.12 for $M = 10$, and from 0.03 to 0.10 for $M = 40$, indicating a trade-off with covertness as higher power increases signal leakage. Increasing *M* reduces both MSE and detection probability, with $M = 40$ achieving up to 26% lower MSE than $M = 10$ at $P_0 = 20 , \text {mW}$, due to better signal aggregation and beamforming precision. Subfigure (b) plots MSE versus channel estimation error variance $\sigma _e^2$ (0 to 0.1). MSE increases with $\sigma _e^2$ for all *M*, e.g., from 0.18 to 0.30 for $M = 10$, and from 0.14 to 0.22 for $M = 40$, reflecting sensitivity to imperfect CSI, which degrades beamforming accuracy. A larger *M* mitigates this impact, with $M = 40$ showing a 27% lower MSE than $M = 10$ at $\sigma _e^2 = 0.1$, highlighting the robustness of massive MIMO against channel errors. These findings emphasize the need for adaptive power allocation and robust CSI estimation in practical deployments.

## Conclusion

In this paper, we presented a cohesive approach for the STAR-RIS-assisted covert ISCCO system by deriving closed-form expressions for reliability (AirComp MSE) and security (covert communication and sensing metrics), which are seamlessly integrated into a novel optimization framework. The expressions directly define the MSE minimization objective and covertness constraints in problem (P1), enabling the joint design of beamformers and STAR-RIS coefficients via a smoothed exact penalty algorithm with CCCP. This integration ensures that reliability and security are not only quantified but also optimized in tandem, achieving a balanced performance as validated by simulations.

Furthermore, simulation results reveal a clear trade-off between covert communication and AirComp MSE, where stricter covertness requirements (smaller $\varepsilon _C$) increase the MSE due to reduced power allocation for data transmission, while looser constraints enhance computational accuracy at the expense of covertness. This trade-off underscores the need for careful parameter tuning in the proposed framework to meet application-specific requirements.

While the proposed system demonstrates strong theoretical performance, its practical implementation faces challenges related to hardware complexity, computational demands, and CSI acquisition. As discussed, advancements in tunable metasurfaces, low-complexity algorithms, and robust optimization techniques are critical for real-world deployment. Future work will focus on addressing these challenges to enhance the system’s applicability in scenarios like secure IoT and autonomous systems.

## Data Availability

Data is provided within the manuscript or supplementary information files
